# RCC1 knockdown sensitizes drug-resistant colorectal cancer to 5-fluorouracil or doxorubicin by impairing DNA repair

**DOI:** 10.20517/cdr.2025.159

**Published:** 2025-11-10

**Authors:** Jing Li, Ya Meng, Xumei Ouyang, Xiaowen Lin, Yangzhe Wu, Hang Fai Kwok

**Affiliations:** ^1^Cancer Centre, Faculty of Health Sciences, University of Macau, Macau 999078, China.; ^2^Guangdong Provincial Key Laboratory of Tumor Interventional Diagnosis and Treatment, Zhuhai People’s Hospital (ZhuHai Clinical Medical College of Jinan University), Zhuhai 519099, Guangdong, China.; ^3^Clinical Center for Immunology and Gut Microecology, Zhuhai Institute of Translational Medicine, Zhuhai People’s Hospital of Beijing Institute of Technology, Zhuhai 519099, Guangdong, China.; ^4^MoE Frontiers Science Center for Precision Oncology, University of Macau, Macau 999078, China.

**Keywords:** RCC1, 5-fluorouracil, doxorubicin, colorectal cancer, chemoresistance, DNA damage response

## Abstract

**Aim:** This study aimed to elucidate the role of regulator of chromosome condensation 1 (RCC1) in colorectal cancer (CRC) progression, as well as its involvement in chemoresistance. We specifically examined how RCC1 knockdown modulates cellular responses, including cell cycle, apoptosis, and senescence induced by 5-fluorouracil (5-FU) or doxorubicin (Doxo) in both parental and drug-resistant CRC cell lines. Additionally, we assessed the potential of RCC1 inhibition as an adjuvant therapeutic strategy to enhance the efficacy of chemoradiotherapy in CRC.

**Methods:** The expression of RCC1 in colon cancer tissues and corresponding adjacent non-cancerous tissues was evaluated through tissue microarrays, and its correlation with characteristics and patient prognosis was also examined. Subsequently, a series of *in vivo* and *in vitro* experiments based on parental and drug-resistant CRC cell lines were conducted to assess the impact of RCC1 knockdown on sensitivity to 5-FU or Doxo. Finally, transcriptomic analysis and subsequent validation assays were performed to explore the underlying molecular mechanisms.

**Results:** RCC1 knockdown significantly enhanced the antitumor efficacy of 5-FU and Doxo in both CRC and drug-resistant CRC cells. In xenograft models, RCC1 knockdown in combination with 5-FU or Doxo suppressed tumor growth with no evident systemic toxicity observed. Transcriptomic profiling and experimental verification revealed that RCC1 knockdown may impair DNA repair by downregulating key repair proteins, thereby leading to more severe and sustained DNA damage.

**Conclusion:** Our results indicate that RCC1 downregulation enhances the responsiveness of both parental and drug-resistant CRC cells to 5-FU and Doxo, highlighting its potential as a therapeutic adjunct to improve clinical outcomes in CRC.

## INTRODUCTION

Colorectal cancer (CRC) continues to impose a significant global health burden due to its high incidence and mortality. As of 2022, CRC was the third most diagnosed cancer, accounting for approximately 9.6% of all new cancer cases, and was the second leading cause of cancer-related deaths worldwide^[1]^. Cancer treatments have advanced considerably over time and continue to improve^[2]^. Despite this, the prognosis for patients with advanced CRC remains poor, with the five-year survival rate estimated to be around 10%^[3,4]^. Currently, 5-fluorouracil (5-FU) continues to be one of the most widely used agents in first-line chemotherapy for CRC. Although cytotoxic treatments such as 5-FU offer some survival benefit, many patients eventually develop resistance during systemic therapy^[5]^. Therefore, enhancing 5-FU chemosensitivity remains a major clinical challenge.

Treatment with 5-FU exerts its anti-cancer effects primarily by interfering with uracil metabolism in tumor cells through multiple mechanisms: (1) as a fluorinated analog of RNA nucleotides, 5-FU metabolites are incorporated into RNA, causing RNA damage, disrupting protein synthesis, and subsequently triggering autophagy and apoptosis^[6,7]^; (2) as a fluorinated analog of DNA nucleotides, 5-FU metabolites can be misincorporated into DNA, directly inducing DNA damage^[8]^; and (3) 5-FU metabolism disrupts deoxynucleotide pools, impairing both DNA synthesis and repair^[8]^. Nevertheless, limited accumulation at tumor sites, associated side effects, and the development of chemoresistance collectively restrict its clinical benefit. Among various mechanisms, the DNA damage response (DDR) plays a pivotal role. Studies have shown that 5-FU-resistant cells preferentially arrest in the G1/S or S phase following drug exposure^[9,10]^, thereby allowing additional time for DNA repair^[11,12]^, which helps mitigate the cytotoxic effects of 5-FU-induced DNA damage. The enhanced DNA repair capacity in chemoresistant cancer cells^[13]^ highlights the potential of therapeutic strategies that simultaneously induce DNA damage and inhibit DDR to counteract resistance.

Regulator of chromosome condensation 1 (RCC1) is a chromatin-associated guanine nucleotide exchange factor for Ran GTPase, playing a crucial role in cell cycle progression and nuclear transport^[14]^. Our previous studies have shown that RCC1 is frequently upregulated in various cancer types compared with adjacent normal tissues, with its upregulation linked to enhanced tumor proliferation and progression^[15]^. Importantly, RCC1 overexpression has been reported to accelerate both cell cycle progression and DDR in CRC cells^[16]^, suggesting that its inhibition could impair these processes and sensitize tumor cells to DNA-damaging agents. Indeed, RCC1 knockdown has been demonstrated to enhance gemcitabine sensitivity in pancreatic ductal adenocarcinoma (PDAC) cells^[17]^. Collectively, these findings suggest that RCC1 overexpression may lead to chemoresistance by accelerating cell cycle progression and DNA repair, highlighting its potential as a compelling therapeutic target for overcoming chemoresistance in cancer therapy.

In this study, we explored how RCC1 affects 5-FU sensitivity in CRC. We first assessed RCC1 expression levels and their association with clinicopathological features using tissue microarray analysis. We then examined the effects of RCC1 knockdown on 5-FU- or doxorubicin (Doxo)-induced cell cycle arrest and apoptosis in both parental and drug-resistant CRC cell lines. In parallel, the impact of RCC1 depletion on tumor progression was evaluated in xenograft models using immunodeficient mice. To uncover potential mechanisms, we quantified DNA damage following drug treatment, examined DDR–related protein changes by Western blot, and performed transcriptomic profiling. Overall, this work provides evidence that targeting RCC1 disrupts DDR and enhances CRC cell sensitivity to 5-FU, providing a potential strategy to counteract chemoresistance.

## METHODS

### Cell lines and CRC tissue microarray

The human colon tissue microarray (HColA180Su19) was purchased from Shanghai Outdo Biotech Co., Ltd. Human CRC cell lines HCT116 and DLD-1 were obtained from the American Type Culture Collection (ATCC). HCT116 cells and their drug-resistant derivatives were cultured in Dulbecco’s Modified Eagle Medium/F12 (DMEM/F12; Gibco, C11330500BT) supplemented with 10% fetal bovine serum (FBS; Procell, 164210-50) and 1% penicillin-streptomycin (Beyotime, C0222). DLD-1 cells were cultured in RPMI-1640 medium (Gibco, C11875500BT) supplemented with 10% FBS. HEK293T cells were cultured in DMEM (Gibco, C11995500BT) containing 10% FBS and 1% penicillin-streptomycin. All cells were maintained at 37 °C in a humidified incubator with 5% CO_2_.

### Lentiviral packaging and establishment of stable cell lines

Details of lentiviral packaging and the generation of RCC1 knockdown or overexpression stable cell lines are provided in the Supplementary Methods.

### Cell proliferation and colony formation assays

For proliferation assays, 4,000 cells per well (adjusted according to the proliferation rate of each cell line) were seeded in 96-well plates in quintuplicate. Cell viability was evaluated at 24, 48, and 72 h using the CCK-8 kit (Beyotime, C0039), and absorbance at 450 nm was measured following a 1-2 h incubation with the reagent.

For colony formation assays, 350 cells per well were seeded into 6-well plates and treated with or without the indicated drug for 24 h. After treatment, the medium was replaced with fresh complete medium, and cells were allowed to grow for an additional 6-10 days until visible colonies formed. Colonies were fixed with 4% paraformaldehyde (PFA; Solarbio, P1110) for 30 min and stained with Crystal Violet Staining Solution (Beyotime, C0121) for another 30 min. Colony areas were quantified using ImageJ software.

### Cell cycle and apoptosis assays

Cells with RCC1 knockdown or overexpression were fixed in prechilled 70% ethanol at -20 °C overnight. After fixation, cells were washed with phosphate-buffered saline (PBS) and treated with RNase A (0.1 mg/mL) at 37 °C for 30 min, followed by staining with propidium iodide (PI; 50 µg/mL) in 0.1% Triton X-100 (Beyotime, P0096) for 15 min at room temperature. Cell cycle distribution was analyzed using a BD FACSCanto II or CYTEK flow cytometer (USA).

Apoptosis was assessed using the FITC-Annexin V Apoptosis Detection Kit (ThermoFisher, 88-8005-74). Cells subjected to single or double staining were fixed in 2% PFA for 5 min and used for compensation controls, while untreated cells served as blank controls. Flow cytometric data were analyzed using FlowJo software (V10.6.2).

### Tumor xenograft mouse models and analyses

All animal studies were approved by the Animal Research Ethics Committee of the University of Macau and conducted in accordance with all relevant ethical regulations (Approval nos. UMARE-001–2019 and UMARE-AMEND-168). Additional details regarding xenograft establishment and analysis are provided in the Supplementary Methods.

### Histology, immunohistochemistry

Tumor tissues harvested from xenograft mice were fixed in 4% PFA and paraffin-embedded for subsequent histological, immunohistochemical (IHC), and immunofluorescent analyses. Detailed procedures are described in the Supplementary Methods.

### Comet assay

Cells (3 × 10^5^ cells/well) were seeded in 6-well plates and treated with or without chemotherapeutic agents for 24 h. The medium was then replaced with fresh medium, and cells were further cultured for 12 h. DNA strand breaks were evaluated using the Comet Electrophoresis Assay Kit (Beyotime, C2041M) following the manufacturer’s protocol. Stained samples were imaged using the Evos FL Auto Imaging System, and tail moments were quantified using the Comet Assay Software Project (CASP).

### Spheroid formation assay

For spheroid formation, single cells were seeded into ultra-low attachment 96-well U-bottom plates (Corning, 7007) at one cell per well or into ultra-low attachment 6-well plates (Corning, 3471) at 1,000 cells per well. Cells were cultured at 37 °C in a humidified incubator containing 5% CO_2_. Spheroid formation was monitored daily using an EVOS imaging system, and both spheroid number and diameter were recorded and analyzed.

The spheroid culture medium consisted of DEME/F12 (Gibco, C11330500BT) supplemented with B27 (50×, serum-free; Gibco), 20 ng/mL epidermal growth factor (EGF), 20 ng/mL basic fibroblast growth factor (bFGF), 4 µg/mL insulin, and 1× N2 supplement.

### Statistical analysis

All statistical analyses were performed using GraphPad Prism (version 5.0). Data are expressed as mean ± standard error of the mean (SEM) from at least two independent experiments. IC_50_ values between independent groups were compared using a two-tailed unpaired *t*-test with Welch’s correction to adjust for unequal variances. Comparisons between two groups were conducted using a two-tailed unpaired *t-*test, and comparisons among multiple groups were performed using one- or two-way analysis of variance (ANOVA) as appropriate. Overall survival was estimated using the Kaplan-Meier method, and differences between groups were assessed with the log-rank test. A *P*-value < 0.05 was considered statistically significant unless otherwise indicated. ns, not significant, ^***^P < 0.001, ^**^P < 0.01, ^*^P < 0.05.

## RESULTS

### RCC1 expression is elevated in colon cancer tissues and correlates with poor prognosis

**Figure 1:**
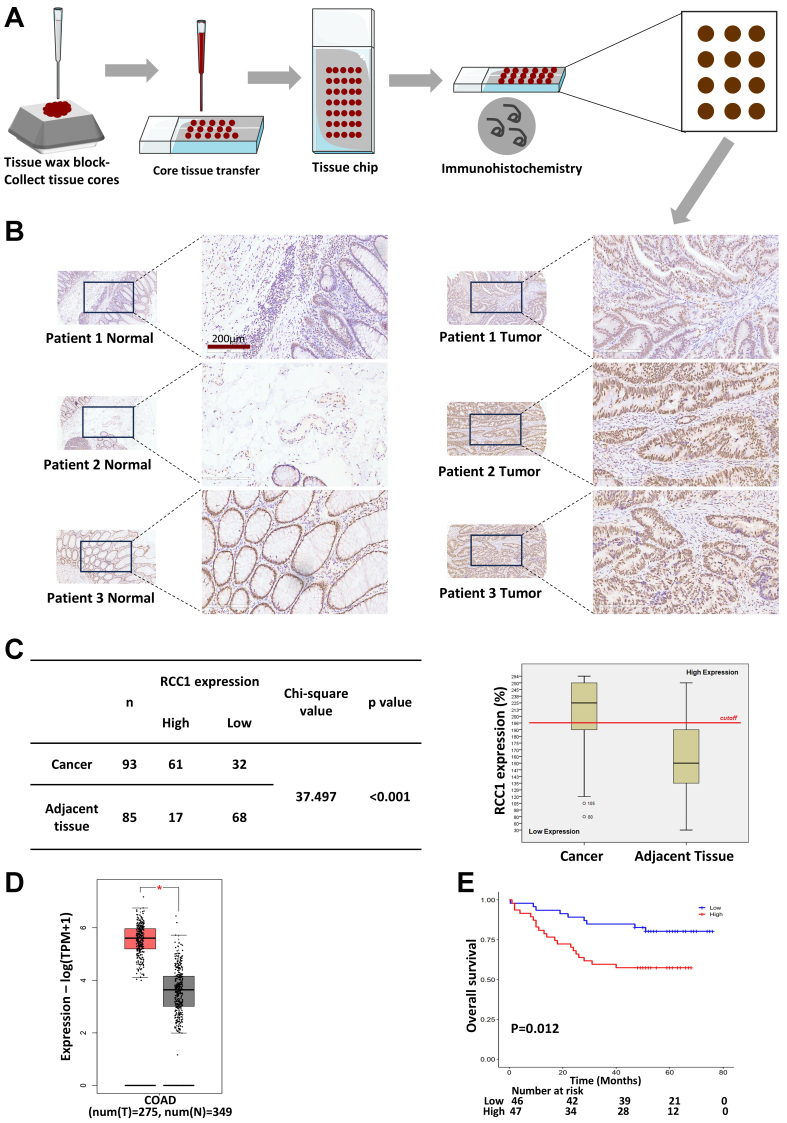
RCC1 is highly expressed in colon cancer tissues. (A) IHC analysis of RCC1 expression in a tissue microarray; (B) Representative images showing RCC1 localization in the tumor and adjacent normal tissues. Scale bar: 200 μm; (C) Distribution of IHC scores with a 200% cutoff defining high *vs.* low expression. The histogram shows higher RCC1 levels in tumor tissues (chi-square test, *P* < 0.001); (D) RCC1 mRNA levels in colorectal tissues from the GEPIA2 database (One-way ANOVA, ^*^P < 0.05); (E) Kaplan-Meier analysis of overall survival based on RCC1 expression (log-rank test, *P* < 0.05). RCC1: Regulator of chromosome condensation 1; IHC: immunohistochemical; COAD: colon adenocarcinom.

To evaluate RCC1 expression in clinical specimens, a tissue microarray containing 93 colon tumor samples and 85 matched adjacent tissues was analyzed by IHC [Figure 1A]. Morphological assessment revealed well-organized crypt and glandular structures with abundant goblet cells in adjacent mucosa, whereas tumor tissues displayed disrupted glandular architecture, nuclear stratification, cellular pleomorphism, and frequent pathological mitoses [Figure 1B].

Quantitative analysis of staining intensity and the proportion of RCC1-positive cells was used to generate a total protein expression score (maximum 300%, calculated as the product of intensity score and positive rate; see Supplementary Methods). A cutoff score of 196% was used to stratify samples into high- or low-expression groups. In tumor tissue, 65.6% of samples exhibited high RCC1 expression compared with only 20% in adjacent normal tissues [Figure 1C, left]. A histogram and chi-square analysis confirmed significantly elevated RCC1 expression in tumor tissues (*P* < 0.001; Figure 1C, right). Consistent results were observed at the transcriptomic level using the GEPIA2 public database [Figure 1D]. Furthermore, Kaplan–Meier analysis demonstrated that high RCC1 expression was associated with significantly worse overall survival in patients with colon cancer (*P* < 0.05; Figure 1E). Multivariate Cox regression analysis further confirmed that RCC1 expression was an independent prognostic factor after adjustment for tumor grade, T stage, and N stage (*P* < 0.05; Supplementary Table 1). No significant correlations were observed between RCC1 expression and other clinicopathological variables (*P* > 0.05; Supplementary Table 2).

### RCC1 knockdown suppresses proliferation and induces G0/G1 arrest in CRC cells

**Figure 2:**
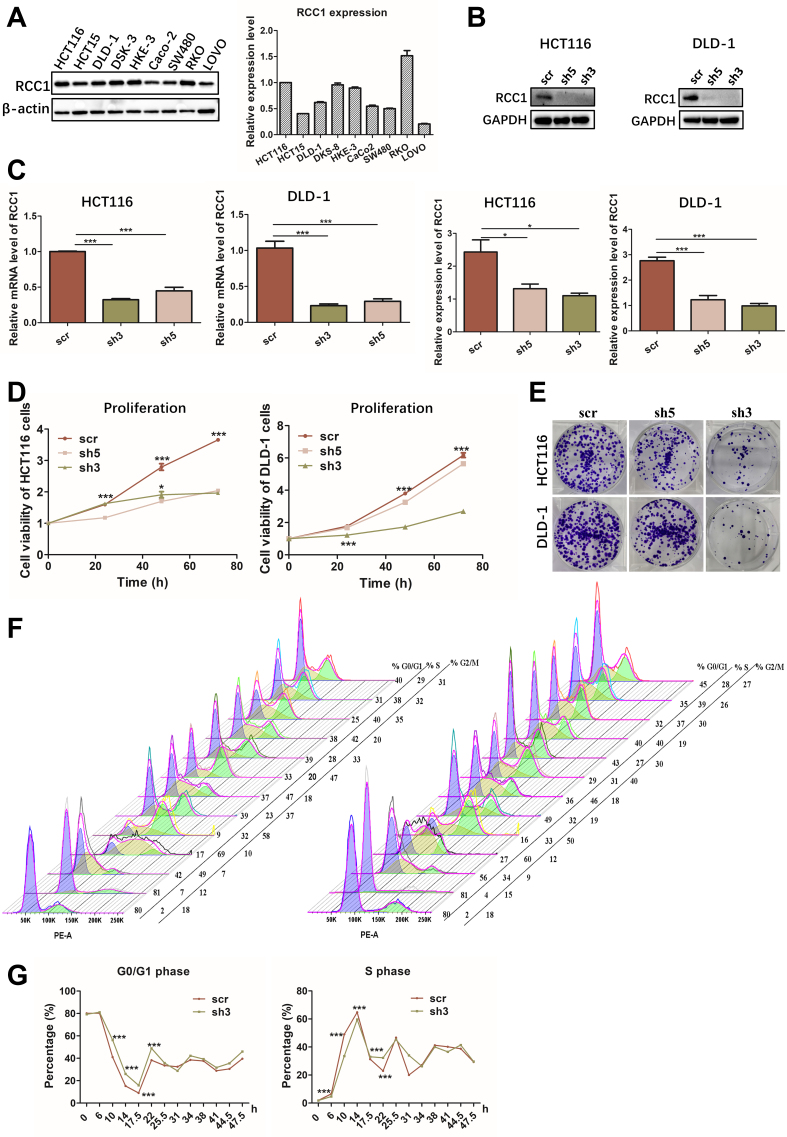
RCC1 knockdown inhibits proliferation and induces G0/G1 phase arrest in CRC cells. (A) Basal RCC1 expression in nine CRC cell lines. Expression in HCT116 was normalized to 1, and relative levels in the other cell lines are shown at right (one-way ANOVA); (B) Top: Western blot analysis of RCC1 protein levels following knockdown in HCT116 cells and DLD-1 cells. Bottom: Quantification showing decreased RCC1 levels in knockdown groups (one-way ANOVA, *P* < 0.001, *P* < 0.05); (C) qRT-PCR analysis of mRNA levels in HCT116 and DLD-1 cells following knockdown (one-way ANOVA, *P* < 0.001); (D and E) CCK-8/MTT proliferation assays and colony formation assays showing reduced growth following RCC1 knockdown (two-way ANOVA, *P* < 0.001); (F and G) Flow cytometry-based cell cycle analysis of synchronized HCT116 cells (two-way ANOVA, *P* < 0.001). ns, not significant, ^***^P < 0.001, ^*^P < 0.05. scr was defined as no knockdown of RCC1 and used as a control. RCC1: Regulator of chromosome condensation 1; CRC: colorectal cancer; ANOVA: analysis of variance; qRT-PCR: quantitative reverse transcription-polymerase chain reaction.

Given the association between RCC1 expression and poor CRC prognosis, we next examined its functional role *in vitro*. Western blot analysis of nine CRC cell lines revealed relatively high basal RCC1 levels in RKO and HCT116 cells. However, based on overall considerations, HCT116 and DLD-1 cells were selected for further analysis [Figure 2A]. RCC1 knockdown was efficiently achieved using shRNA, as confirmed by western blotting and quantitative reverse transcription-polymerase chain reaction (qRT-PCR, Figure 2B and C). Functional assays demonstrated that RCC1 knockdown significantly suppressed proliferation and colony-forming ability in both HCT116 and DLD-1 cells [Figure 2D and E, Supplementary Figure 1A], with shRCC1-3 producing the strongest inhibitory effect (one-way ANOVA, *P* < 0.001).

To explore the mechanism underlying this growth inhibition, we synchronized HCT116 cells in G0/G1 using lovastatin and monitored cell cycle re-entry. Flow cytometry revealed that RCC1-knockdown cells exhibited a delayed G1-to-S transition compared to controls, maintaining a higher proportion of cells (80%-89%) in G0/G1 and showing a slower decline in S-phase entry (two-way ANOVA, *P* < 0.001; Figure 2F and G, Supplementary Figure 1B). Western blot analysis of cyclin expression further confirmed that cell cycle progression was attenuated following RCC1 knockdown [Supplementary Figure 1C and D]. Collectively, these results suggest that RCC1 knockdown impedes CRC cell proliferation primarily by enforcing G0/G1 arrest. Scramble (scr) was defined as no knockdown of RCC1 and used as a control.

### RCC1 knockdown suppresses the growth of 5-FU- and Doxo-resistant CRC cells

Because chemoresistance poses a major clinical challenge, we next investigated the role of RCC1 in drug-resistant CRC models. Stable 5-FU- and Doxo-resistant HCT116 cell lines were established through sequential high-dose and dose-escalation drug selection [Supplementary Figure 2A]. Compared with parental HCT116 cells, the resistant cells exhibited markedly higher IC_50_ values (~30 µM for 5-FU and ~950 nM for Doxo), reduced proliferation but enhanced colony-forming capacity, and elevated expression of P-gp and RCC1 [Supplementary Figure 2B-F], confirming successful model generation.

**Figure 3:**
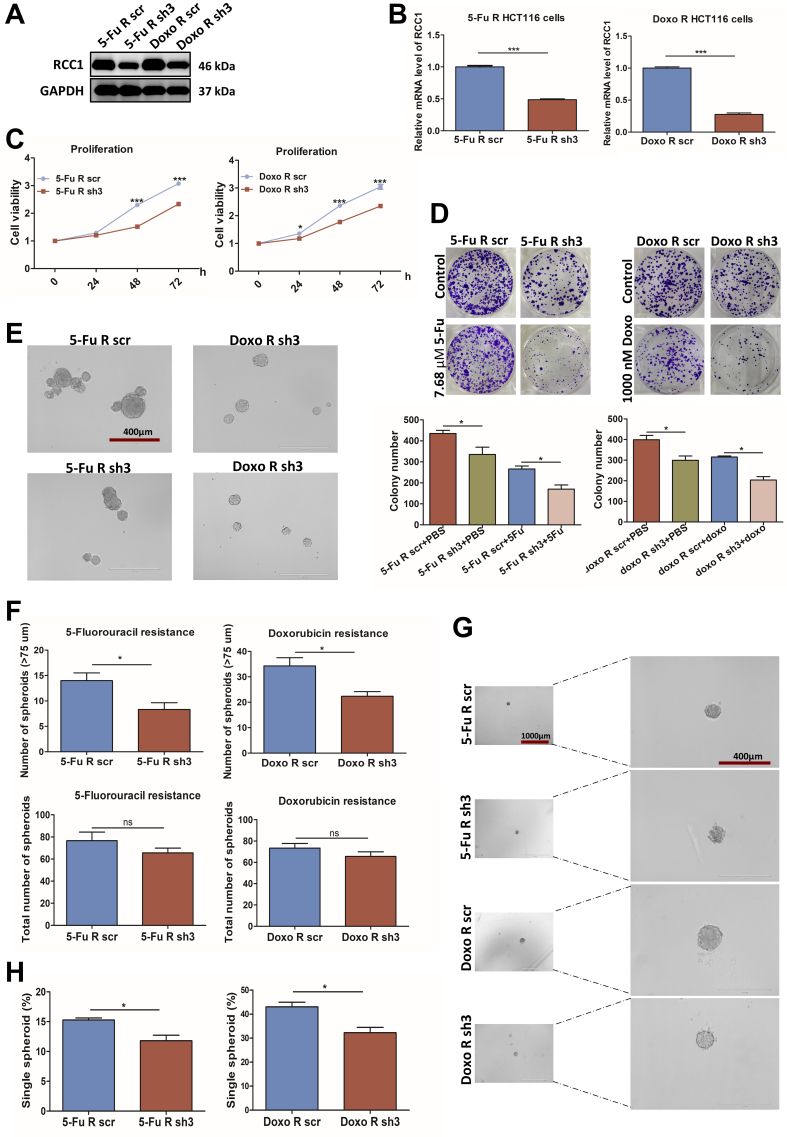
RCC1 knockdown impairs proliferation in 5-FU- and Doxo-resistant HCT116 cells. (A and B) Validation of RCC1 knockdown in 5-FU- and Doxo-resistant HCT116 cells by western blotting (A) and qRT-PCR (B) (two-tailed unpaired *t*-test, *P* < 0.001); (C and D) Reduced proliferation (CCK-8 assay) and colony formation following RCC1 knockdown (two-way ANOVA, *P* < 0.001, *P* < 0.05); (E) Representative images of 5-FU- or Doxo-resistant CRC spheroids cultured for 5 days. Scale bar: 400 µm; (F) Total spheroid counts and number of spheroids with diameters > 75 μm (two-tailed unpaired *t*-test, *P* < 0.05); (G) Representative images of 5-FU- or Doxo-resistant CRC single spheroids cultured for 8 days. Scale bar: 400 μm; (H) Quantitative analysis of total single spheroids (two-tailed unpaired *t*-test, *P* < 0.05). ns, not significant; ^***^P < 0.001; ^*^P < 0.05. RCC1: Regulator of chromosome condensation 1; 5-FU: 5-fluorouracil; Doxo: doxorubicin; qRT-PCR: quantitative reverse transcription-polymerase chain reaction; ANOVA: analysis of variance; CRC: colorectal cancer.

RCC1 knockdown in the resistant cells was confirmed by western blotting and qRT-PCR [Figure 3A and B] and led to significant inhibition of proliferation and colony formation [Figure 3C and D].

In spheroid assays, RCC1 depletion lowered the proportion of large spheroids (> 75 μm) and reduced the efficiency of single-cell spheroid formation [Figure 3E-H], indicating impaired self-renewal capacity. Together, these findings suggest that RCC1 is essential for maintaining the growth potential of drug-resistant CRC cells.

### RCC1 knockdown enhances chemosensitivity by promoting cell cycle arrest and apoptosis

**Figure 4:**
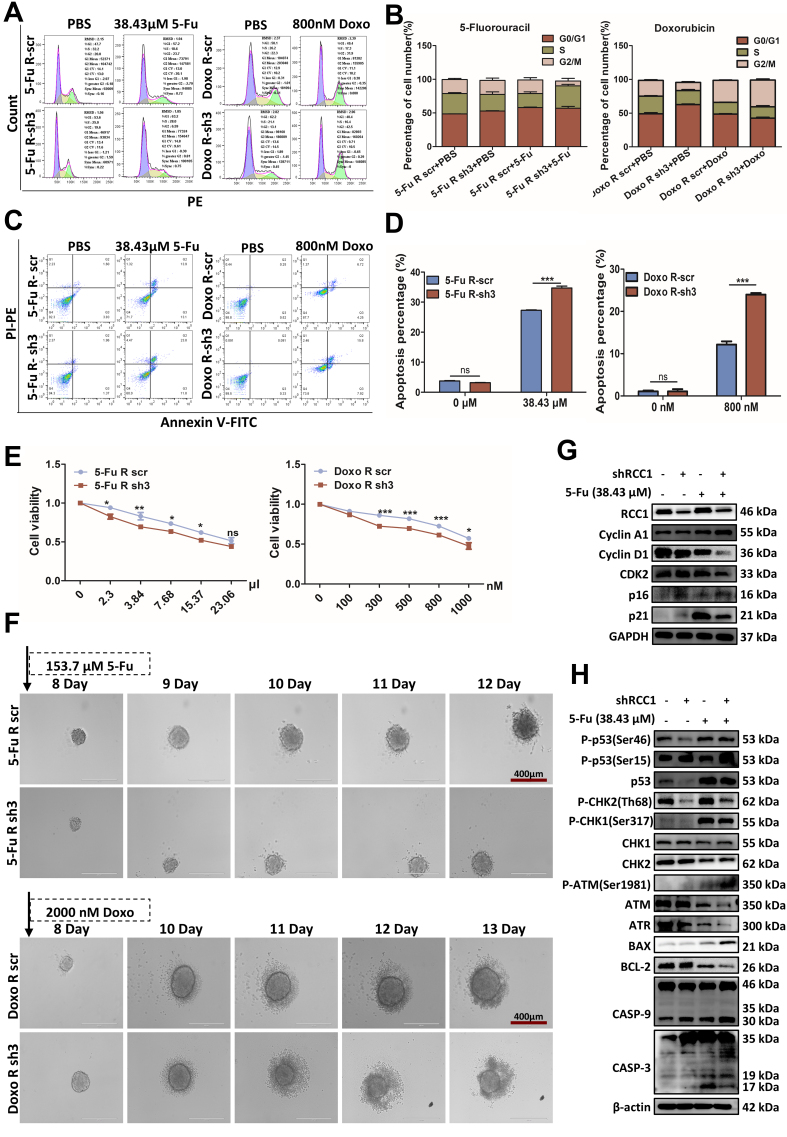
RCC1 knockdown sensitizes drug-resistant CRC cells to chemotherapy. (A) Cell cycle distribution in 5-FU- and Doxo-resistant HCT116 cells assessed by flow cytometry after 48-h treatment with 38.43 µM 5-FU or 800 nM Doxo, with or without RCC1 knockdown; (B) Statistical analysis of cell cycle phase proportions (one-way ANOVA); (C) Apoptosis in 5-FU- and Doxo-resistant HCT116 cells after 48-h treatment with 38.43 µM 5-FU or 800 nM Doxo, with or without RCC1 knockdown; (D) Statistical analysis of apoptotic cell populations (two-way ANOVA); (E) Cell viability (CCK-8 assay) of 5-FU- and Doxo-resistant HCT116 cells after 48-h treatment with various drug concentrations, with or without RCC1 knockdown (two-way ANOVA); (F) Tumor spheroid disruption assessed in drug-resistant HCT116 cells after 5-day treatment with 153.7 µM 5-FU or 2,000 nM Doxo; (G) Western blot analysis of cell cycle-related proteins after treatment with 38.43 µM 5-FU. GAPDH was used as the loading control; (H) Western blot analysis of cell cycle- and apoptosis-related proteins following treatment with 800 nM Doxo. β-actin served as the loading control. ns, not significant; ^***^P < 0.001; ^**^P < 0.01; ^*^P < 0.05. RCC1: Regulator of chromosome condensation 1; CRC: colorectal cancer; 5-FU: 5-fluorouracil; Doxo: doxorubicin; ANOVA: analysis of variance; GAPDH: glyceraldehyde-3-phosphate dehydrogenase.

To investigate whether RCC1 knockdown sensitizes CRC cells to chemotherapy, we analyzed cell cycle distribution and apoptosis in drug-resistant HCT116 cells using flow cytometry. Drug concentrations for resistant CRC cells were selected based on their respective IC_50_ values: ~30 μM for 5-FU-resistant HCT116 cells and ~1,000 nM for Doxo-resistant HCT116 cells [Supplementary Figure 2B]. As depicted in Figure 4, RCC1 knockdown alone induced G0/G1 arrest. When combined with 5-FU or Doxo, it further increased arrest in S and/or G2/M phases, depending on the drug. Specifically, RCC1 knockdown with 38.43 µM 5-FU resulted in both S and G0/G1 arrest (*P* < 0.01), whereas combination with 800 nM Doxo led to G2/M arrest (*P* < 0.001, Figure 4A and B). These cell cycle changes were accompanied by increased p16 and p21 levels [Figure 4G and Supplementary Figure 3A], which mediate G1-S phase transition and G2/M phase arrest. Consistent results were observed in other CRC cells, where RCC1 knockdown caused G0/G1 arrest in both HCT116 and DLD-1 cell lines [Supplementary Figures 4A and 5A], accompanied by increased p16 levels [Supplementary Figure 4B and C]. Following treatment with 5-FU or Doxo, cell cycle arrest occurred in G0/G1, S and G2/M phases, respectively, in HCT116 and DLD-1 cells [Supplementary Figures 4A and 5A]. Drug concentrations for non-resistant CRC cells were also based on IC_50_ values: ~10 µM (HCT116) and ~13 µM (DLD-1) for 5-FU, and ~300 nM (HCT116) and ~626 nM (DLD-1) for Doxo [Supplementary Figure 6A]. RCC1 knockdown also potentiated chemotherapy-induced apoptosis, as evidenced by increased Annexin V positivity and upregulation of pro-apoptotic proteins BAX, cleaved CASP-3, and CASP-9, along with downregulation of the anti-apoptotic protein BCL-2 [Figure 4C, D, H; Supplementary Figure 3B and C]. CCK8 assays confirmed enhanced cytotoxicity across a range of drug concentrations [Figure 4E], reflected by decreased IC_50_ values in the RCC1 knockdown group [Supplementary Figure 3D and E]. Similar results were observed in HCT116 and DLD-1 cells [Supplementary Figures 4D and E, 5B-D, 6B and C]. In 3D spheroid models, RCC1 knockdown accelerated spheroid disintegration in response to high-dose 5-FU (153.7 µM) or Doxo (2,000 nM) [Figure 4F]. Mechanistically, these effects were associated with increased activation of key kinases involved in cell cycle arrest and apoptosis, including p-ATM/ATR, p-CHK1/p-CHK2, p53, and phosphorylated p53 at Ser46 and/or Ser15 [Figure 4H and Supplementary Figure 3B], indicating an enhanced DDR. Taken together, these findings suggest that RCC1 knockdown sensitizes both parental and drug-resistant CRC cells to 5-FU and Doxo by activating the ATM/ATR-CHK1/CHK2-p53-p21 signaling axis, thereby promoting cell cycle arrest and apoptosis.

### RCC1 knockdown augments chemotherapy efficacy *in vivo*

**Figure 5:**
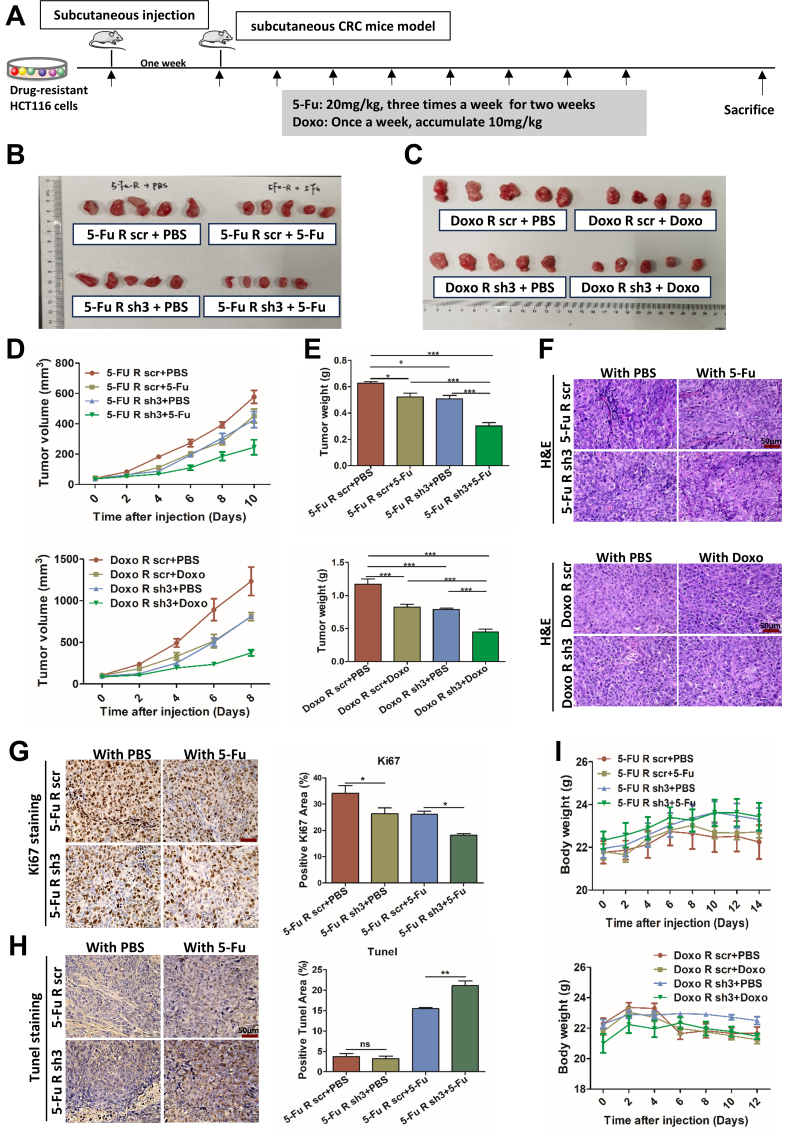
RCC1 knockdown enhances the antitumor effects of 5-FU or Doxo *in vivo*. (A) Schematic overview of the experimental design. Drug-resistant CRC cells were subcutaneously injected into nude mice, followed by systemic drug administration after tumor formation. Mice were randomly divided into four groups. For the 5-FU-resistant group: scr + PBS (*n* = 5), sh3 + PBS (*n* = 5), scr + 5-FU (*n* = 5), sh3 + 5-FU (*n* = 5), (20 mg/kg, 3×/weeks for 2 weeks). For the Doxo-resistant group: scr + PBS (*n* = 5), sh3 + PBS (*n* = 5), scr + Doxo (*n* = 5), sh3 + Doxo (*n* = 5) (10 mg/kg cumulative, 1×/week); (B and C) Representative images of excised subcutaneous tumors in the 5-FU- or Doxo-resistant models after treatment; (D) Tumor volume measurements during treatment (two-way ANOVA); (E) Final tumor weights across all groups (one-way ANOVA); (F) Representative H&E staining of tumor sections from each group. Scale bar: 50 μm; (G) Ki-67 immunohistochemistry (left) and quantification of Ki-67-positive cells (right) using ImageJ (one-way ANOVA). Scale bar: 50μm; (H) TUNEL staining (left) and quantification of apoptotic cells (right) across groups. Scale bar: 50 μm; (I) Body weight monitoring of mice during treatment to assess tolerability (two-way ANOVA). ns, not significant; ^***^P < 0.001; ^**^P < 0.01; ^*^P < 0.05. RCC1: Regulator of chromosome condensation 1; 5-FU: 5-fluorouracil; Doxo: doxorubicin; CRC: colorectal cancer; PBS: phosphate-buffered saline; scr: scramble; ANOVA: analysis of variance.

To validate these findings *in vivo*, we established subcutaneous xenograft models using either parental or drug-resistant HCT116 cells, with or without RCC1 knockdown, and treated the mice systemically with 5-FU or Doxo [Figure 5A and Supplementary Figure 7A]. Tumors were harvested after treatment for analysis [Figure 5B and C, Supplementary Figure 7B]. RCC1 knockdown significantly reduced tumor volume [Figure 5D and Supplementary Figure 7C] and weight [Figure 5E and Supplementary Figure 7D], indicating suppressed CRC growth. When combined with 5-FU or Doxo, RCC1 knockdown further decreased tumor burden compared to chemotherapy alone, suggesting enhanced drug sensitivity. Histological analysis by H&E staining was performed to evaluate tissue morphology [Figure 5F and Supplementary Figure 7E], and Ki-67 immunohistochemistry was used to assess cell proliferation. Tumors in the RCC1 knockdown plus 5-FU/Doxo groups showed markedly reduced Ki-67 expression compared to those in the control or monotherapy groups [Figure 5G, Supplementary Figures 3F and 7F]. Furthermore, TUNEL staining revealed increased apoptosis in tumors treated with the combination therapy relative to 5-FU or Doxo alone [Figure 5H, Supplementary Figures 3G and 7G]. These findings demonstrate that RCC1 knockdown enhances the antitumor effects of 5-FU or Doxo in drug-resistant CRC. To evaluate potential treatment-related toxicity, the mouse body weight was monitored throughout the study. Only minor, non-significant weight loss was observed in all groups [Figure 5I and Supplementary Figure 7H], suggesting that RCC1 knockdown combined with chemotherapy is well tolerated *in vivo*.

### RCC1 knockdown potentiates 5-FU/Doxo-induced DNA damage and impairs DDR

**Figure 6:**
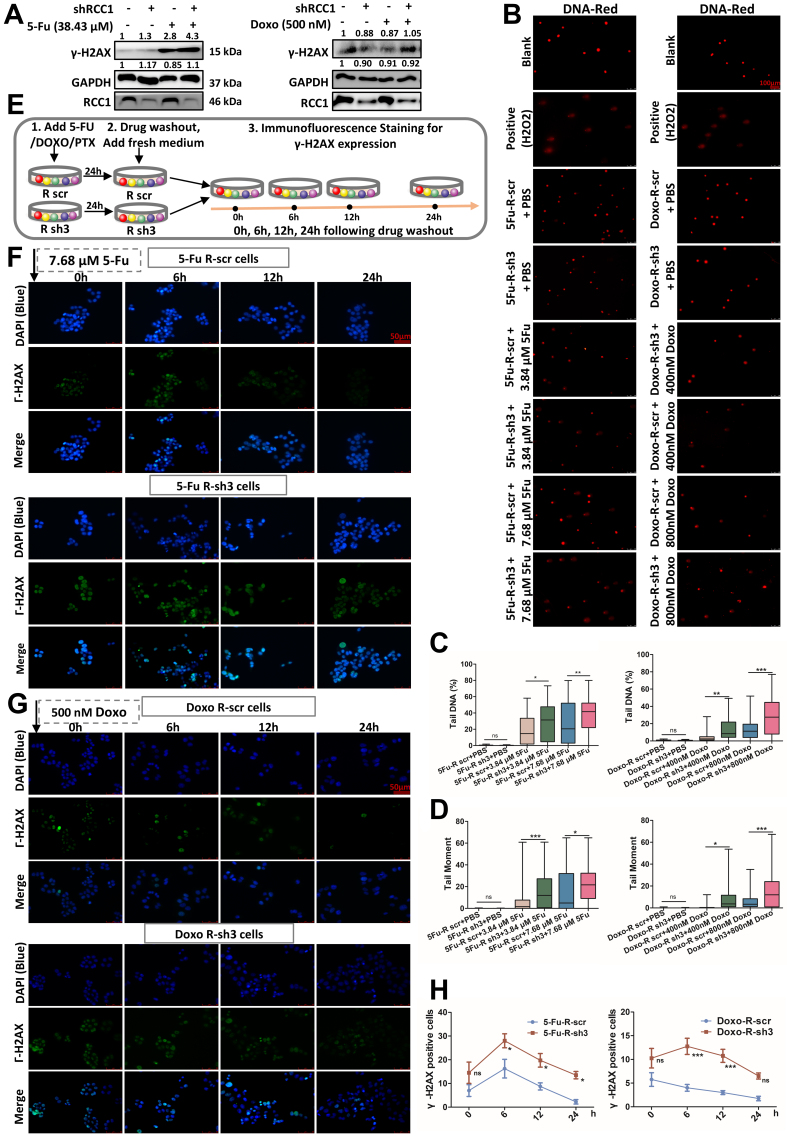
RCC1 knockdown enhances DNA damage and impairs DDR in drug-resistant CRC cells. (A) Western blot analysis of γ-H2AX expression in drug-resistant CRC cells treated with 38.43 μM 5-FU or 500 nM Doxo for 48 h, with or without RCC1 knockdown for 48 h; (B) Representative comet assay images of drug-resistant CRC cells treated with the indicated drugs for 24 h. Scale bar: 100 μm; (C and D) Quantification of DNA damage by comet assay, including percentage of DNA in tail (C) and tail moment (D) across treatment groups (one-way ANOVA); (E) Schematic of the experimental design for monitoring DNA damage repair dynamics via γ-H2AX immunofluorescence at 0, 6, 12, and 24 h post-drug withdrawal; (F and G) Representative immunofluorescence images showing time-dependent changes in γH2AX-positive cells after treatment with 7.68 μM 5-FU (F) or 500 nM Doxo (G) in drug-resistant CRC cells with or without RCC1 knockdown. Scale bar: 50 μm; (H) Quantification of γH2AX-positive cells at 24 h after drug removal in the indicated groups (two-way ANOVA). ns, not significant; ^***^P < 0.001; ^**^P < 0.01; ^*^P < 0.05. RCC1: Regulator of chromosome condensation 1; DDR: DNA damage response; CRC: colorectal cancer; 5-FU: 5-fluorouracil; Doxo: doxorubicin; ANOVA: analysis of variance.

Given that enhanced DDR is a hallmark of chemoresistant cancer cells, we investigated whether RCC1 contributes to this process in CRC. Following treatment with 5-FU or Doxo for 48 h, RCC1 knockdown markedly elevated γ-H2AX expression in both parental and drug-resistant CRC cells compared to drug treatment alone or control cells [Figure 6A and Supplementary Figure 8A]. To further evaluate DNA integrity at the single-cell level, we conducted comet assays. These assays revealed that RCC1 knockdown significantly induced DNA damage, as indicated by an increased proportion of DNA in the tail and tail moment [Figure 6B-D, Supplementary Figure 8B-E]. Moreover, tail length extended in a dose-dependent manner, indicating enhanced DNA damage upon RCC1 silencing. Collectively, these results demonstrate that RCC1 knockdown amplifies DNA damage induced by 5-FU or Doxo.

To determine whether this effect was linked to compromised DNA repair capacity, we monitored DDR kinetics using γ-H2AX immunofluorescence at defined intervals (0, 6, 12, and 24 h) after drug withdrawal [Figure 6E]. To compare damage induction and repair rates under RCC1 knockdown, cells were treated with drug concentrations below the IC_50_, selected to ensure all cells remained adherent during treatment. While γ-H2AX levels declined within 24 h in the drug-only group, they remained persistently elevated in the RCC1 knockdown plus 5-FU/Doxo groups, indicating impaired DNA repair [Figure 6F-H]. A similar trend was observed in parental CRC cells at a single time point [Supplementary Figure 8F and G].

These findings suggest that RCC1 depletion not only augments chemotherapy-induced DNA damage but also delays its resolution, thereby sensitizing cells to genotoxic stress. Notably, γ-H2AX levels remained above baseline for an extended period in the RCC1 knockdown plus 5-FU/Doxo groups [Figure 6F-H], consistent with persistent DNA damage.

### RCC1 knockdown impairs DNA repair by downregulating nuclear repair genes

**Figure 7:**
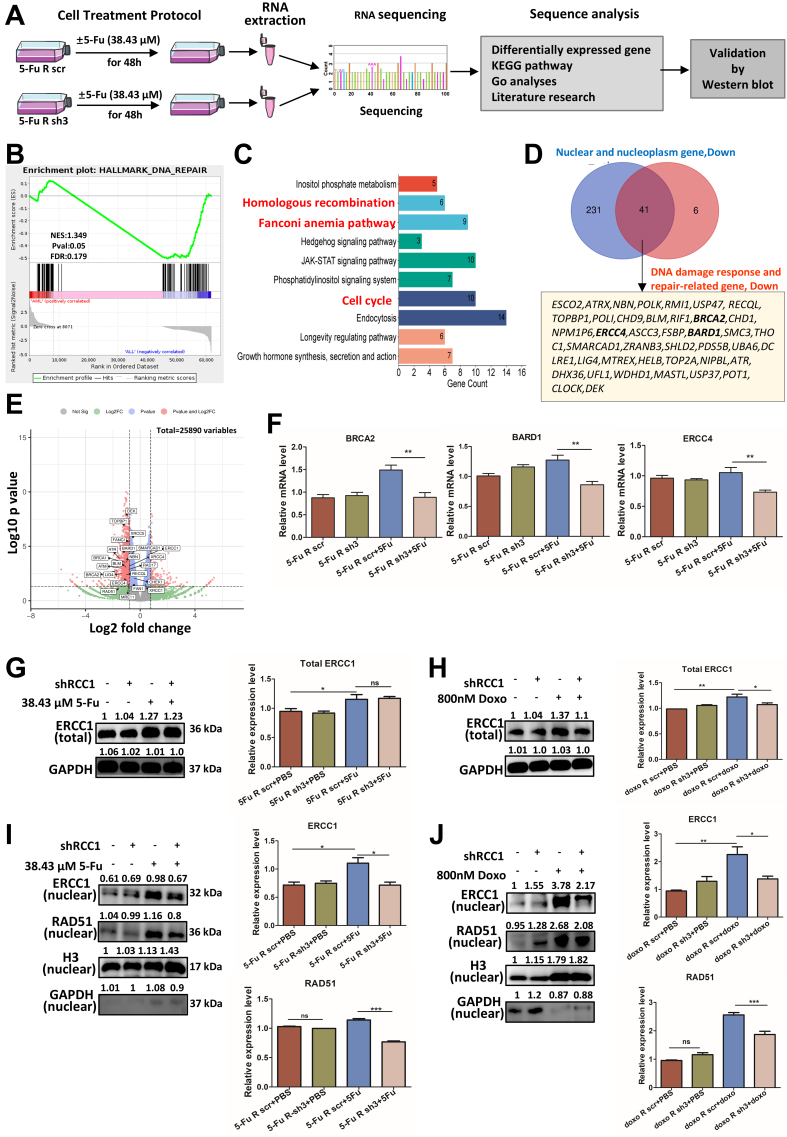
RCC1 knockdown disrupts the expression and nuclear localization of DNA repair proteins. (A) Schematic overview of the workflow used to investigate molecular changes in 5-FU-resistant HCT116 cells with or without RCC1 knockdown (*n* = 2 per group); (B) GSEA showing significant downregulation of DNA repair pathways in the RCC1 knockdown + 5-FU group; (C) KEGG pathway enrichment analysis of differentially expressed genes, highlighting downregulated pathways including HR, FA, and cell cycle pathways; (D) GO analysis focusing on nuclear and DNA repair-related genes identified 41 potential downregulated targets (fold change < 0.5, RCC1 knockdown + 5-FU *vs.* control + 5-FU); (E) Volcano plot of differentially expressed genes in the RCC1 knockdown plus 5-FU treatment group; DNA repair-related genes are highlighted; (F) qRT-PCR analysis of *ERCC4*, *BRCA2*, and *BARD1* RNA levels, showing significant reductions upon RCC1 knockdown (one-way ANOVA, *P* < 0.01); (G and H) Western blot analysis of total ERCC1 protein levels (one-way ANOVA); GAPDH served as an internal control; (I and J) Western blot analysis of nuclear ERCC1 and RAD51 levels (one-way ANOVA); H3 was used as a loading control. ns, not significant; ^***^P < 0.001; ^**^P < 0.01; ^*^P < 0.05. RCC1: Regulator of chromosome condensation 1; 5-FU: 5-fluorouracil; GSEA: gene set enrichment analysis; KEGG: Kyoto Encyclopedia of Genes and Genomes; HR: homologous recombination; FA: Fanconi anemia; GO: Gene Ontology; qRT-PCR: quantitative reverse transcription-polymerase chain reaction; ANOVA: analysis of variance; GAPDH: glyceraldehyde-3-phosphate dehydrogenase.

To elucidate the molecular basis for the impaired DNA repair observed following RCC1 knockdown, we conducted RNA-seq analysis in 5-FU-resistant HCT116 cells with or without 5-FU treatment [Figure 7A]. Notably, gene set enrichment analysis (GSEA) revealed a significant downregulation of the “DNA repair” pathway in the RCC1 knockdown group [Figure 7B]. Additionally, Kyoto Encyclopedia of Genes and Genomes (KEGG) pathway analysis identified significant suppression of key repair-related pathways, including homologous recombination (HR), Fanconi anemia (FA), and cell cycle pathways [Figure 7C]. Gene Ontology (GO) analysis of 412 differentially expressed genes localized to the nucleus and nucleoplasm in the 5-FU-resistant sh3 + 5-FU group showed that 379 were downregulated. By intersecting 272 downregulated nuclear genes with 47 downregulated DDR-related genes, we identified 41 potential target genes exhibiting more than a 2-fold reduction in expression [Figure 7D]. These genes were enriched in HR and interstrand crosslink (ICL) repair processes, as visualized in a volcano plot [Figure 7E]. Taken together, these results suggest that RCC1 knockdown disrupts nuclear DNA repair networks, particularly those essential for HR and ICL repair, thereby promoting persistent DNA damage.

To validate these findings, we examined the transcript levels of selected repair genes. qRT-PCR confirmed that BRCA2, BARD1, and ERCC4 mRNA levels were significantly reduced upon RCC1 knockdown [Figure 7F]. At the protein level, total ERCC1 expression remained largely unchanged [Figure 7G and H], whereas nuclear levels of RAD51 and ERCC1 - two critical mediators of HR and nucleotide excision repair (NER) - were substantially decreased in RCC1-knockdown cells following 5-FU or Doxo treatment [Figure 7I and J].

In summary, RCC1 knockdown exacerbates chemotherapy-induced DNA damage by impairing the expression and nuclear localization of DNA repair proteins, thereby sensitizing both CRC and drug-resistant CRC cells to 5-FU and Doxo.

## DISCUSSION

Despite the continued clinical use of 5-FU or Doxo, resistance remains a major barrier to effective CRC therapy^[8]^. Enhancing chemosensitivity in resistant CRC cells is therefore a critical goal, but clinically effective sensitizers remain lacking. Advances in genomic and transcriptomic profiling, single-cell analysis, and multi-omics approaches have revealed mutations and expression changes associated with resistance, while CRISPR-based functional screens can directly identify causal resistance genes^[18,19]^. These high-throughput technologies provide powerful tools to understand chemoresistance and guide the development of new therapeutic strategies. In this study, we demonstrated that RCC1 knockdown significantly improved the antitumor effects of 5-FU and Doxo in both parental and drug-resistant CRC cell lines and animal models, *in vitro* and *in vivo*. Mechanistically, RCC1 knockdown promoted sustained DNA damage, disrupted key DDR signaling, and induced potent cell cycle arrest and apoptosis. These findings nominate RCC1 as a promising therapeutic target for restoring chemosensitivity in CRC.

Although the role of RCC1 in CRC remains underexplored, prior studies have implicated it in cancer progression and prognosis. For example, it was reported that elevated RCC1 expression correlates with poor prognosis in liver-metastatic CRC, independent of clinicopathologic features^[20]^. Our results align with this, showing that high RCC1 protein expression is an independent unfavorable prognostic factor in colon cancer. Similar to observations in other cancers^[17,21-23]^, we found that RCC1 knockdown induced G0/G1 arrest and suppressed proliferation in both parental and resistant CRC cells. RCC1, a chromatin-associated guanine nucleotide exchange factor, generates the Ran-GTP gradient, which is essential for cell cycle regulation and chromatin dynamics, affecting nucleocytoplasmic transport, spindle assembly, and cell cycle progression^[24]^. Notably, elevated RCC1 expression regulates the RanGTP gradient, accelerates cell cycle transitions, and strengthens the cellular ability for DNA repair^[16,17]^. Consistent with reports that RCC1 depletion inhibits Skp2 nuclear import, thereby blocking the G1–S transition^[23]^, we also found that RCC1 regulates cell cycle progression via control of transport dynamics, thus influencing CRC cell proliferation*.* Together, these results suggest that elevated RCC1 may increase intracellular Ran-GTP levels, driving faster cell cycle transitions. Such rapid proliferation, coupled with enhanced repair, improves tolerance to genomic damage, contributing to both proliferative capacity and chemotherapy resistance.

Growing evidence highlights the pivotal role of RCC1 in mediating resistance to chemo- or radiotherapy. In pancreatic cancer, RCC1 silencing sensitizes tumors to gemcitabine^[17]^, while in glioblastoma, methylation at arginine 214 (R214) of RCC1 reduces RanGTP levels, enhancing radiosensitivity^[25]^. In our study, RCC1 knockdown enhanced cell cycle arrest and apoptosis following 5-FU or Doxo treatment, accompanied by modulation of key regulatory proteins, including increased pro-apoptotic markers, reduced anti-apoptotic Bcl-2, and altered cell cycle regulators. Notably, RCC1 knockdown also suppressed ATM and ATR expression while increasing phosphorylation of p53 at Ser15 and/or Ser46, modifications associated with DNA damage-induced cell cycle arrest, apoptosis, or senescence^[26]^. Specifically, Ser46 phosphorylation of p53 is linked to the induction of apoptosis^[27-30]^, while Ser15 phosphorylation is strongly associated with both cell cycle arrest and senescence via the p53-p21 pathway^[28]^.

The ATM/ATR–CHK1/CHK2–p53 axis plays a critical role in maintaining genome integrity, and its dysregulation frequently contributes to therapy resistance^[31,32]^. In our study, RCC1 knockdown reduced total ATM, ATR, CHK1, and CHK2 protein levels. Phosphorylation dynamics were site-specific: CHK2 Thr68 phosphorylation decreased, while CHK2 Ser516 and CHK1 Ser345 modification increased. Prior reports have indicated that CHK2 is necessary for p53-dependent apoptosis^[33-37]^, with Ser516 phosphorylation promoting apoptosis^[35,36,38]^ and Thr68 phosphorylation linked to proliferation and chemoradiotherapy response^[39,40]^. Additionally, CHK1 Ser317 phosphorylation - implicated in cell cycle re-entry and resistance mechanisms^[41]^ - was observed in RCC1-knockdown cells. Collectively, these findings suggest that RCC1 knockdown may modulate the DDR signaling cascade to favor tumor suppression via apoptosis and growth arrest.

The chemotherapeutic agent 5-FU primarily acts by incorporating into DNA and RNA, disrupting DNA replication and repair^[8]^, and inducing cell S-phase arrest^[9,10]^. We observed that RCC1 knockdown significantly increased 5-FU–induced cell cycle arrest in both S and G0/G1 phases, and led to accumulation of γ-H2AX, a marker of DNA double-strand breaks (DSBs) and apoptosis^[42,43]^. DSBs are primarily repaired via non-homologous end joining (NHEJ) and HR^[13]^. HR is particularly critical in repairing replication stress- or replication blocking agents-induced breaks^[44]^ and is frequently activated in cancer cells to maintain genomic stability during high proliferation^[45]^. Targeting HR-associated DDR proteins (e.g., BRCA1/2, RAD51, BARD1) has emerged as a strategy to overcome chemoresistance^[46,47]^.

Our transcriptomic analysis revealed that RCC1 knockdown suppressed multiple HR-related genes, including BRCA2, BARD1, and RAD51, under 5-FU treatment. BRCA2 protects stalled replication fork^[48-50]^ and facilitates RAD51 loading at DNA breaks to initiate strand invasion^[51]^, while BARD1 forms a complex with BRCA1 to support DNA end resection and HR initiation^[52-54]^. RAD51, a central recombinase, drives homologous pairing and strand exchange^[55]^, and its inhibition has been shown to induce senescence and suppress tumor growth^[56]^. Thus, by disrupting HR, RCC1 knockdown may diminish CRC cells’ ability to repair DSBs and tolerate chemotherapy-induced replication stress. Additionally, we observed suppression of the FA pathway in RCC1-depleted cells following 5-FU treatment. The FA pathway, a form of break-induced replication (BIR), repairs stalled replication forks via ICL repair^[57]^ and is associated with therapy resistance^[58]^. Downregulation of both FA and HR pathways may synergistically impair replication fork recovery, leading to genomic instability and cell death. As the effect of inhibiting HR or ICL shown by RCC1, we wondered whether the combination of RCC1 inhibitors with PARP inhibitors or other DDR-targeting agents to overcome drug resistance in a “synthetic lethal” manner.

We also observed reduced nuclear localization of ERCC1, a key endonuclease involved in NER, ICL repair, and DSB repair via the ERCC1–XPF complex^[51]^ (XPF also known as ERCC4). Previous studies have shown that high doses of oxaliplatin trigger proteasome-mediated degradation of ERCC1, indicating that ERCC1 abundance determines whether cells undergo DNA repair or apoptosis in response to DNA-damaging agents^[59]^. Knockout of ERCC4 leads to cytoplasmic retention of ERCC1, indicating that their nuclear localization and function are interdependent^[60]^. Although ERCC1 mRNA levels were unchanged, nuclear ERCC1 protein declined upon RCC1 knockdown. This discrepancy may result from post-translational regulation or impaired nuclear import. RCC1 regulates nucleocytoplasmic transport via the RanGTP gradient^[16]^, and methylation of RCC1 at R214 disrupts Ran activation and RCC1-mediated mitosis and nucleocytoplasmic transport in glioblastoma^[25]^. Thus, RCC1 may facilitate nuclear trafficking of DDR proteins such as ERCC1, and its depletion may impair these processes.

In summary, our findings demonstrate that RCC1 knockdown significantly enhances the chemosensitivity of both conventional and drug-resistant CRC cells to 5-FU and Doxo. Combining RCC1 suppression with either chemotherapeutic agent markedly promotes cell cycle arrest and apoptosis, surpassing the effects of monotherapy. Mechanistically, this enhanced response is associated with impaired nuclear transport and downregulation of key DDR proteins, resulting in deficient DNA repair and sustained DNA damage. These disruptions in genome maintenance pathways ultimately resensitize CRC cells to chemotherapy. Taken together, our study highlights RCC1 as a potential therapeutic target and supports a combinatorial strategy that may improve treatment outcomes in patients with drug-resistant CRC.

In addition to the impact of RCC1 expression levels on drug resistance, it remains unclear whether alterations in RCC1 at the translational or post-translational modification levels also contribute to resistance. Furthermore, low-dose chemotherapy regimens were used *in vivo* to minimize toxicity. While these regimens effectively demonstrate sensitization, they may underestimate the full therapeutic potential. Therefore, targeted delivery systems - such as nano-polymeric carriers, which have shown efficacy in preclinical breast cancer studies^[61]^ - may offer a promising approach to enhance tumor-selective uptake of RCC1 inhibitors and chemotherapeutics while minimizing adverse effects.

## DECLARATIONS

### Availability of data and materials

Data are provided within the manuscript or Supplementary Materials. Materials are available from the corresponding author upon reasonable request.

### Copyright

© The Author(s) 2025.
