# Supplementary material for: RCC1 knockdown sensitizes drug-resistant colorectal cancer to 5-fluorouracil or doxorubicin by impairing DNA repair
Source: Cancer Drug Resist. 2025 Nov 10;8:58. doi: 10.20517/cdr.2025.159 (PMC12635986; doi:10.20517/cdr.2025.159)
Supplement: Supplementary file 1 [file cdr-8-58-SupplementaryMaterials.pdf]

Supplementary Materials

RCC1 knockdown sensitizes drug-resistant colorectal cancer to 5-fluorouracil or doxorubicin by impairing DNA repair

Jing Li[1,2], Ya Meng[2], Xumei Ouyang[2], Xiaowen Lin[2], Yangzhe Wu[2,3], Hang Fai Kwok[1,4]

[1]Cancer Centre, Faculty of Health Sciences, University of Macau, Macau 999078, China.
[2]Guangdong Provincial Key Laboratory of Tumor Interventional Diagnosis and Treatment, Zhuhai People's Hospital (ZhuHai Clinical Medical College of Jinan University), Zhuhai 519099, Guangdong, China.
[3]Clinical Center for Immunology and Gut Microecology, Zhuhai Institute of Translational Medicine, Zhuhai People's Hospital of Beijing Institute of Technology, Zhuhai 519099, Guangdong, China.
[4]MoE Frontiers Science Center for Precision Oncology, University of Macau, Macau 999078, China.

Correspondence to: Prof. Hang Fai Kwok, Cancer Centre, Faculty of Health Sciences, University of Macau, Macau 999078, China. E-mail: hfkwok@um.edu.mo; Prof. Yangzhe Wu, Clinical Center for Immunology and Gut Microecology, Zhuhai Institute of Translational Medicine, Zhuhai People's Hospital of Beijing Institute of Technology, Zhuhai 519099, Guangdong, China. Email: wu_yz@bitzh.edu.cn

Supplementary Methods

Cell cycle synchronization

A total of 1 × 10^6 HCT116 scr cells and HCT116 sh3 cells were seeded separately into 100-mm petri dishes. After 24h of incubation in complete medium containing 15 μmol/L of Lovastatin (Selleck.cn, S2061), the medium was replaced with fresh culture medium containing 1.5 mM of mevalonate (MCE, HY-113071), equivalent to a tenfold concentration of lovastatin. Beginning from the time of mevalonate addition, cells were harvested at 4-hour intervals, and then fixed in 70% alcohol overnight at 4 °C.

Tumor xenograft mouse models and analyses

A subcutaneous tumor xenograft model was employed in this study. Male BALB/c nude mice (6-8 weeks old) were purchased from BesTest Biotechnology Co., Ltd. (Zhuhai, China). Briefly, tumor cells (1 × 10^6 per side per mouse) were resuspended in a 1:1 mixture of FBS and Matrigel (Corning, 354234), and then transplanted subcutaneously the flanks of the mice. Body weight was recorded every other day. Once the tumor could be palpated, the tumor volumes were recorded every other day using calipers, and calculated using the formula: $V = L \times W^2 \times \pi/6$. In the meantime, the mice were divided into groups and given intraperitoneal injections of drugs. After the treatment, the mice were euthanized, and the tumors will be removed and divided into 2 parts, one was fixed in 4% paraformaldehyde (PFA), and the other was rapidly snap-frozen in liquid nitrogen.

Group information:

CRC subcutaneous xenograft models: For 5-FU-resistant group, including scr+ PBS (n=5), sh3+PBS (n=5), scr+5-FU (n=5), sh3+5-FU (n=5), (20mg/kg, 3×/weeks,2 weeks). For Doxo-resistant group, including, scr + PBS (n=5), sh3+PBS (n=5), scr +Doxo (n=5), sh3+Doxo (n=5) (10 mg/kg cumulative, 1×/week).

Drug-resistant CRC subcutaneous xenograft models: For 5-FU-resistant group, including scr + PBS (n=5), sh3+PBS (n=5), scr +5-FU (n=5), sh3+5-FU (n=5), (20mg/kg, 3×/weeks,2 weeks). For Doxo-resistant group, including, scr + PBS (n=5), sh3+PBS (n=5), scr +Doxo (n=5), sh3+Doxo (n=5) (10 mg/kg cumulative, 1×/week).

Extraction of cell protein and Western blot

Total cellular proteins were extracted using RIPA lysis buffer (Thermo Scientific, 89900) contains protease inhibitors (Roche, 4693132001), followed by centrifugation at 12,000 rpm for 25 min at 4 °C. The BCA kit (23225, Thermo Scientific) was used to quantified the protein concentration according to the manufacturer's instructions. Equal amounts of protein were resolved on 7.5%-12.5% SDS–polyacrylamide gels (prepared through Omni-Easy™ One-step Staining PAGE Gel Rapid Preparation Kit, PG221, PG222, PG223) and transferred to polyvinylidene fluoride (PVDF) membrane (Millipore, ISEQ00010). Membranes were blocked in QuickBlock ™ Western Blocking Buffer (Byeotime, P0252) for 40 min at room temperature, followed by overnight incubation with primary antibodies at 4 °C. After three 10min washes with TBST (Beyotime, ST673) for 10 min each time, membranes were incubated with secondary antibodies for 1 h at room temperature. The binding signals were quantified using Omega Lum C (Aplegen, US). GAPDH or β-actin was used as the internal loading control.

For nuclear protein isolation, the nuclear protein and cytoplasmic protein extraction kit (Beyotime, P0028) was used for the extraction of nuclear protein, the procedure of the experiment is according to the instructions. After the extraction of cell proteins, the nuclear proteins are detected by Western blotting analysis, as described above. H3 was used as the loading control.

Antibody list:

Cyclin D1 (0186-1-Ig), Cytochrome c (10993-1-AP), p53 (10442-1-AP), P-gp (22336-1-AP), Phospho-P53 (Ser15) (67826-1-Ig), Phospho-P53 (Ser46) (28960-1-AP), CHK1 (25887-1-AP), CHK2 (13954-1-AP), P16-INK4A (10883-1-AP), p21 (10355-1-AP), ATM (27156-1-AP), ATR (19787-1-AP), Cyclin B1 (55004-1-AP), Cyclin A2 (18202-1-AP), Cyclin E1 (11554-1-AP), Caspase 3/p17/p19 (19677-1-AP), Caspase 9/p35/p10 (10380-1-AP) and RCC1 (22142-1-AP) antibody were purchased from Proteintech.

p21 Waf1/Cip1 (12D1) (CST,2947), GAPDH (CST, 2118), β-Actin (4967), phospho-Chk2 (Ser516) (2669), BAX (D2E11) (CST, 5023), Cleaved Caspase-9 (Asp353) (9505) and BCL-2 (D17C4) (3498) were purchased from CST.

RAD51 (F1110), H3 (F0057), ERCC1 (F1083), Phospho-Chk2 (Thr68) (F0243), Phospho-Chk1 (Ser345) (F0242) and Phospho-Chk1 (Ser317) (F0495) were purchased from Selleck.

Phospho-Histone H2A.X (S139) (T56572S) was purchased from Abmart.

mRNA extraction and quantitative reverse transcription PCR (qRT-PCR)

Total RNA of HCT116 cells was extracted using RNeasy Kit (74136, Qiagen, Germany), The High-Capacity cDNA Reverse Transcription Kit (4368814, Thermo Scientific) was used to reverse transcribe RNA to cDNA in a Gradient thermal cycler (C1000 Touch, Bio-Rad). Gene-specific primers were designed based on sequences obtained from the NCBI database. Quantitative real-time PCR was performed using Real-Time PCR System (Thermo Scientific, ABI PRISM 7300 Plus real-time PCR system).

The qRT-PCR reaction system contains (20 μL system):

PowerUp™ SYBR™ Green Master Mix (ThermoScientific)	10 μL
Primer	2 μL
cDNA	2 μg
ddH2O	contributing a total volume of 20 μL.

The PCR cycling conditions were as follows: initial denaturation at 95 °C for 2 min, followed by 40 cycles of denaturation at 95 °C for 15 s and annealing/extension at 60 °C for 30 s. A melting curve analysis was subsequently performed with steps of 95 °C for 15 s, 60 °C for 60 s, and 95 °C for 15 s. The $2^{-\Delta\Delta Ct}$ method was used to analyze differences of expression levels among the amplified genes. GAPDH was used as the internal reference gene.

Primer:

RCC1:	F: GACCAGAAAACCCGACCAG
	R: CTCCATCACATTCTCACCCAG
GAPDH:	F: GACCACAGTCCATGCCATCAC
	R: TCCACCACCCTGTTGCTGTAG
BRCA2:	F: GAAGCGTGAGGGGACAGATT
	R: ATTGGTACAGCGGCAGAGTC
BARD1:	F: TCTCCATACCTGCCACCAGT
	R: AGCAGAGTAACCTCCCCACA
ERCC4:	F: GATTTAATTACTGGCATCTTGGTGT
	R: AGAGGCGCAAGATGAATGCT

Histology, immunohistochemistry

After embedding the isolated tissues with paraffin wax, the paraffin-tissue was cut into sections with a thickness of 5 μm using the ASP6025 Tissue processor (Leica). Paraffin-embedded sections were utilized for hematoxylin and eosin (H&E) staining and immunohistochemistry (IHC) analysis. The process is as follows:

For H&E staining, tissue section was put in a 60 °C oven, baking the wax for 1 h. The tissue sections were then deparaffinized using 100% dimethylbenzene and rehydrated through the graded ethanol series 100%, 100%, 95%, 85%, 70%, followed by distilled water, for 7, 5, 5, 5, 5, 5 min. After rehydration, the tissue sections were stained with hematoxylin for 4min and then rinsed with running tap water for 10min, followed by differentiated with ethanol hydrochloride for 3 s, rinsed with tap water for 10min, and stained with eosin for 30 s. The tissue sections were then dehydrated with 100%, 100%, 100% ethanol (3 changes, 5 min each) and permeated with 100, 100, 100% dimethylbenzene (3 changes, 10 min each). Finally, the slices are sealed with a neutral resin and left to dry naturally before being photographed under a microscope.

For IHC, after rehydration, antigen retrieval treatment was done in pH 6.0, 0.01 mol/L sodium citrate buffer in an autoclave at 121 °C for 2 min Endogenous peroxidase activity was blocked by treatment with 3% hydrogen peroxide for 20 min at room temperature. The tissue sections were then washed in PBS and blocked with 10% BSA for 30 min. Sections were then incubated with primary antibodies overnight at 4 °C in a humidified chamber. Following three washes with PBS, sections were incubated with horseradish peroxidase HRP-conjugated secondary antibody for 10-60 min at room temperature. After additional PBS washes, visualization was performed using DAB solution (ZhongShan Biotechnology), followed by Hematoxylin re-staining for 1 min and dehydrate and cleared for 5~10 min each, Finally, slides were dehydrated, cleared, sealed with neutral resin, air dried, and imaged.

Determination of staining intensity: The entire field of view of the tissue point was observed under a low magnification microscope, and it was divided into 5 grades: negative (0 point, 0), low (0.5 point, 0.5+), low to medium strength (1 point, 1+), moderate (2 points, 2+) and high (3 points, 3+). (Note: If there are both moderate and high in the tissue, it is generally recorded as 2-3.

Determination of positive staining rate: first observe the entire field of view of the tissue points under a low magnification microscope, then select 3 fields of view with different staining intensities for interpretation under a high magnification microscope,

and randomly record 100 cells in each field of view, and then record them. The percentage of positive cells in 100 cells is X1%, and the percentage of positive cells in the other two fields of view is X2% and X3% in the same principle. Finally, the positive rate of staining in this tissue spot is the average of X1%, X2% and X3%.
Total score: the product of "staining intensity score" and "staining positive rate", (0-300%).
Survival analysis grouping: Nuclei: the total score [0, 200%] is the low expression group, and [200%, 300%] is the high expression group.

Immunofluorescence
For immunofluorescence assays, 2000 cells were seeded into a 96-well plate and treated with the indicated drug for 24 h. DNA damage was then evaluated using the DNA Damage (by γ-H2AX Immunofluorescence) Assay Kit (Beyotime, C2035S), following the manufacturer's instructions. Images were captured using Evos FL Auto Imaging System, and ImageJ was used for image quantification and analysis.

Lentiviral packaging and the establishment of stable cell lines
The overexpression plasmid and lentiviral constructs of RCC1-shRNA and their matched scramble vectors were purchased from Horizon. HEK293T cells were plated in 6-well plates at a density of 7.5×10^5/well. According to the instructions of the Lipo3000 kit (Invitrogen, L3000015), the plasmids were transfected into HEK293T cells, and viral supernatants were collected after two days of incubation. Next, the virus was then filtered through a 45-μm PES filter (Sartorius, 16533), then aliquoted and stored at -80 °C for later use.
Colorectal cancer cell lines HCT116, DLD-1 and drug-resistant HCT116 cells were seeded in 6-well plate at the density of 4×10^5 cells per well before lentivirus infection. Once the confluence of cells reached around 70%, lentiviruses were added in the presence of polybrene (8 μg/mL for knockdown, 2 μg/mL for overexpression) (Selleck, E1299-25 mg) to infect target cells. Subsequently, fresh medium containing puromycin (ThermoFisher, A1113803) was used to select for successfully transduced cells. Following selection, the cells were harvested for mRNA and protein analysis to verify the establishment of the stable cell lines before expansion. Finally, the shSCR cells, shRCC1 cells, RCC1-OE cells, drug-resistant shSCR cells and drug-resistant shRCC1 cells, were obtained for further studies.

Supplementary Table 1. Univariate and multivariate analyses of the factors influencing OS with a Cox proportional hazard model

Variables	Univariate analysis				Multivariate analysis			
	***P* value**	**HR**	**95%CI Lower limit**	**Upper limit**	***P* value**	**HR**	**95%CI Lower limit**	**Upper limit**
RCC1 expression	**0.016**	2.631	1.196	5.786	**0.046**	2.305	1.014	5.238
Age	0.305	0.679	0.324	1.422				
Sex	0.751	1.126	0.541	2.342				
Tumor size	0.374	0.712	0.337	1.506				
Grade stage	**0.005**	2.888	1.377	6.057	0.104	0.1909	0.876	4.163
TNM stage	0.001	3.688	1.709	7.957	0.913	0.000	0.000	1.455E+63
T stage	**0.011**	3.016	1.286	7.071	0.113	2.031	0.845	4.879
N stage	**<0.001**	4.159	1.926	8.981	0.901	17984.184	0.000	1.432E+71
M stage	0.052	3.280	0.998	10.888				

TNM stage: tumor-node-metastasis classification; OS: overall survival; HR: hazard ratio; CI: confidence interval.

Supplementary Table 2. Correlation between RCC1 expression and clinicopathological characteristics

	variables	*RCC1* expression		total	χ^2	*P* value
		low	high			
**Age (year)**	≤65	15	32	47	0.348	0.555
	>65	17	28	45		
**Sex**	male	15	30	45	0.045	0.833
	Female	17	31	48		
**Grade**	II	29	48	77	2.099	0.147
	III	3	13	16		
**T stage**	T2/T3	16	25	41	0.692	0.405
	T4	16	36	52		
**N stage**	N0	21	37	58	0.221	0.638
	N1/N2	11	24	35		
**M stage**	M0	31	57	88	0.486	0.486
	M1	1	4	5		
**TNM stage**	I/II	21	35	56	0.596	0.440
	III/IV	11	26	37		
**Tumor size**	≤5.3cm	17	30	47	0.082	0.775
	>5.3cm	15	30	45		

TNM stage: tumor-node-metastasis classification. Chi-square test, *P*>0.05.

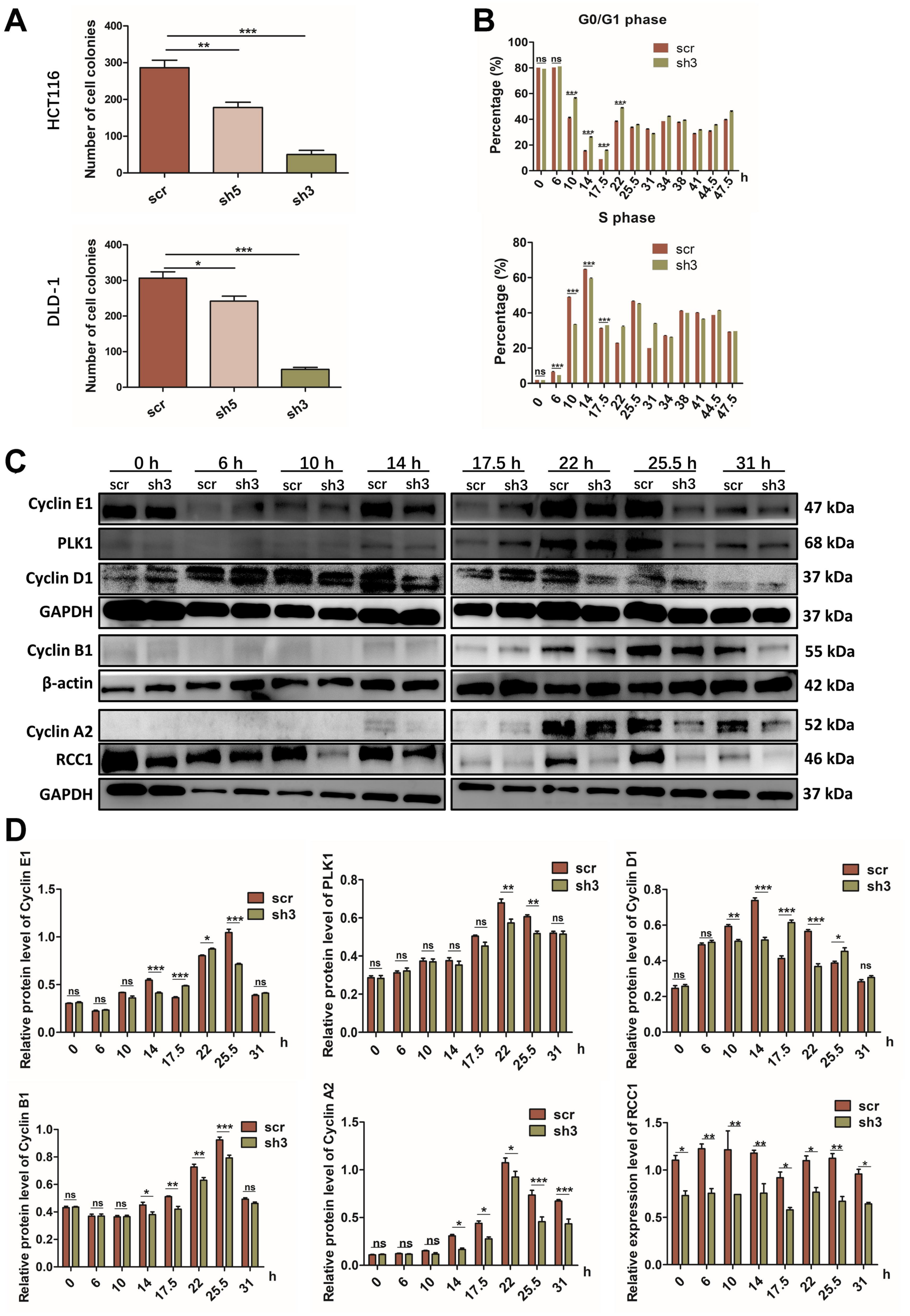

Supplementary Figure 1. (A) Quantification of the number of colonies formed after knockdown of RCC1 in CRC cells (One-way ANOVA); (B) Quantification of the proportion of cells in each phase of the cell cycle for a long time after knockdown of RCC1 in HCT166 cells (Two-way ANOVA); (C) Cell cycle-related proteins analysis

by western blot. GAPDH and β-actin serves as loading control; (D) Quantification of relative protein content from Supplementary Figure 1C. ns, not significant; ***P<0.001; **P<0.01; *P<0.05. Scramble (scr) was defined as no knockdown of RCC1 and was used as a control.

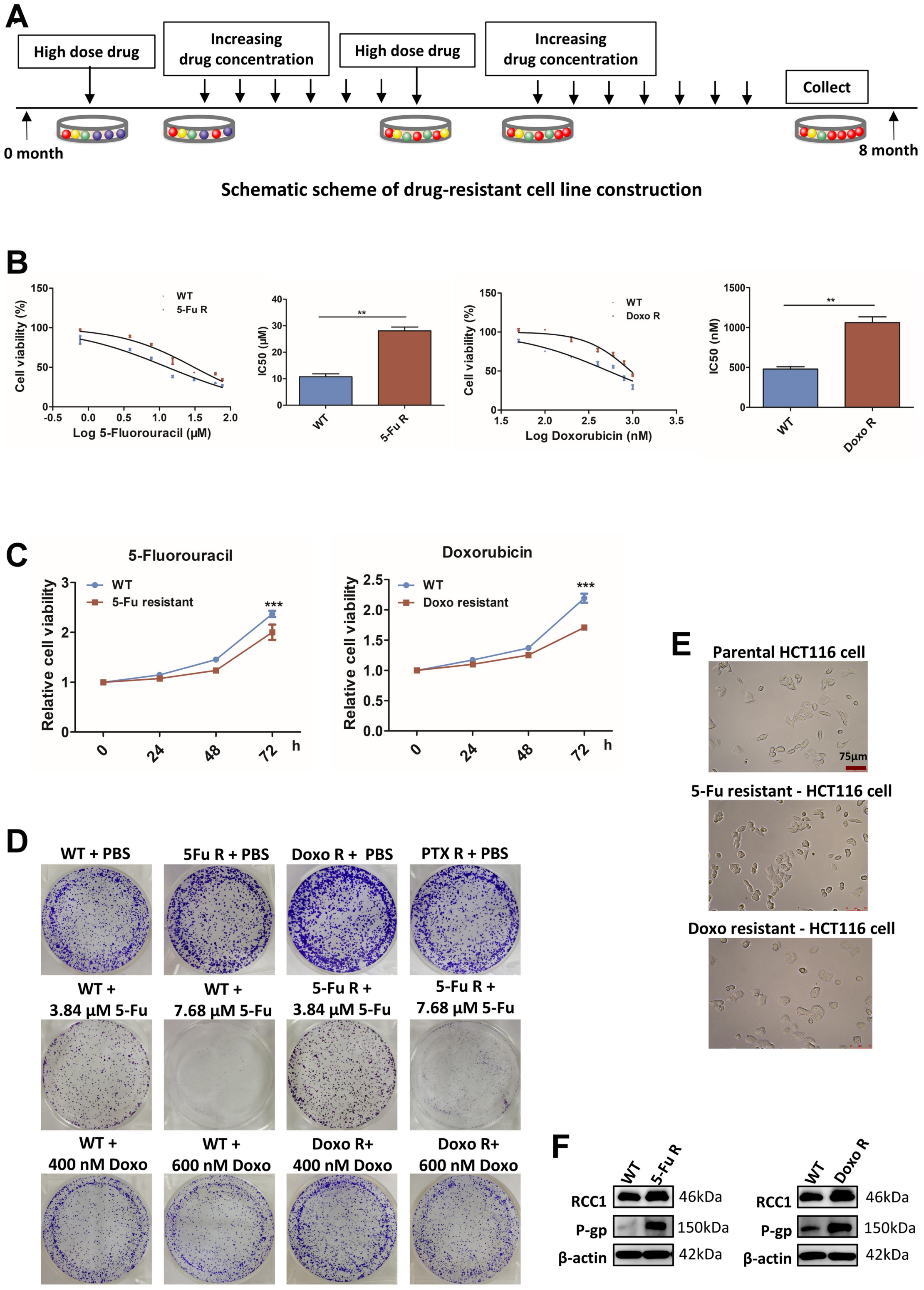

Supplementary Figure 2. Establishment of drug-resistant colorectal cancer cell lines. (A) Schematic scheme of drug-resistant cell line construction, a method that is combination of high-dose drug shock and increasing drug concentration to generate drug-resistant colorectal cancer cell lines; (B) Comparison of IC_{50} between parental

and drug-resistant HCT116 cells (Two-tailed unpaired *t* test with Welch's correction); (C) The proliferation of parent cells and 5-Fu-/Doxo-resistant HCT116 cells was evaluated by CCK8 assay of cell viability (Two-way ANOVA); (D) The colony formation of parent cells and 5-Fu-/Doxo-resistant HCT116 cells was detected after indicated drug treatment; (E) Comparison of cell morphology between parent cells and 5-Fu-/Doxo-resistant HCT116 cells. Scale bar: 75 μm; (F) Comparison of P-gp expression between parental and drug-resistant HCT116 cells. GAPDH was used as internal loading controls. ns, not significant; ***$P<0.001$; **$P<0.01$; *$P<0.05$.

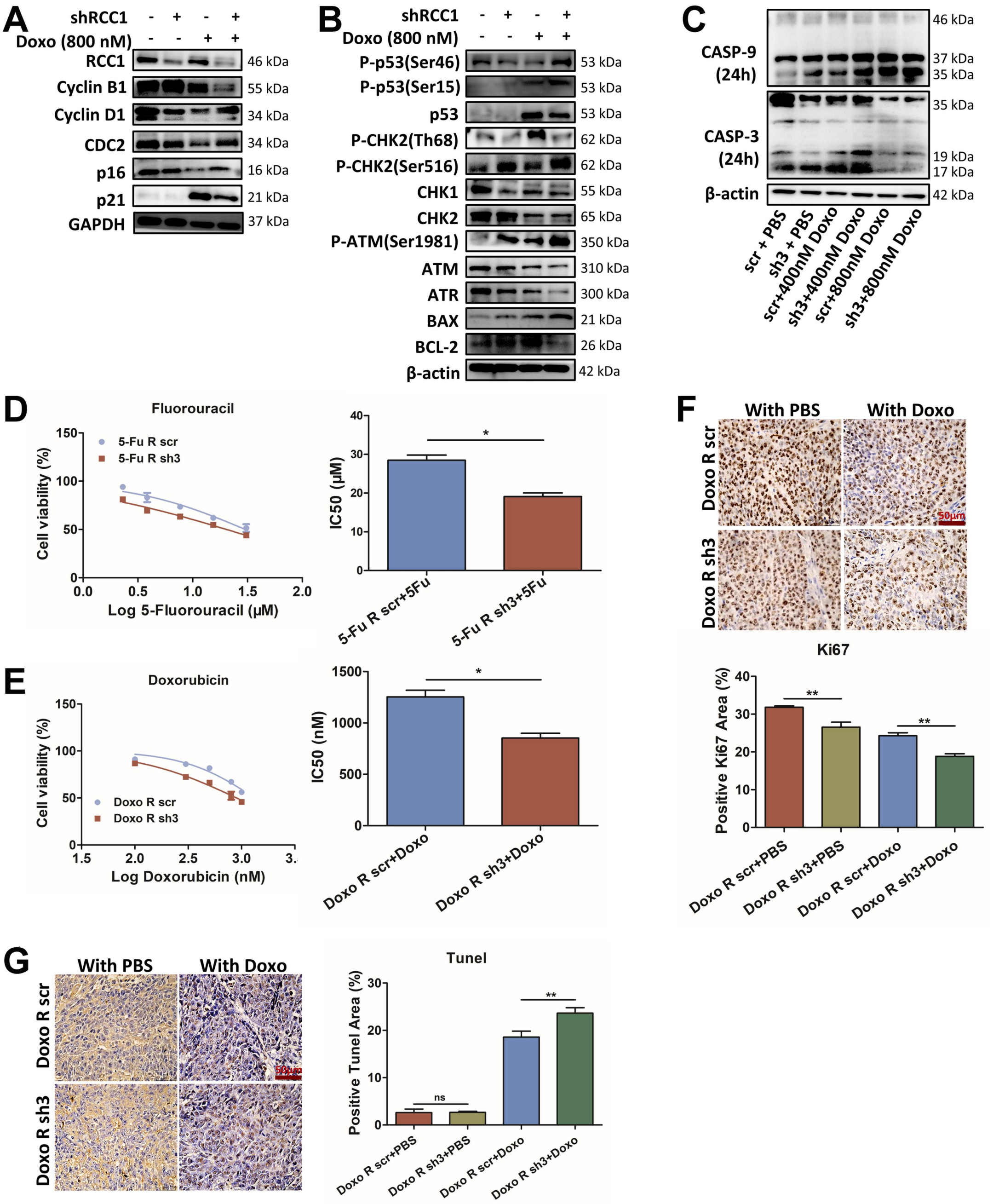

Supplementary Figure 3. (A-C) Western blotting analysis was performed to examine the expression of cell cycle- and apoptosis-related proteins after treating with 800nM Doxorubicin in Doxo-resistant HCT116 cells. GAPDH or β-actin was used as internal loading controls; (D and E) The IC_{50} of drug-resistant HCT116 cells with or without RCC1 knockdown (Two-tailed Unpaired *t* test with Welch's correction); (F) Top: Representative Ki-67 IHC staining images of tumor tissue sections from each group of Doxo-resistant CRC-bearing mice. Scale bar: 50 μm. Bottom: Quantification of Ki-67 positivity using ImageJ (One way ANOVA); (G) Left: Representative TUNEL IHC staining images of tumor tissue sections from each group of Doxo-resistant

CRC-bearing mice. Scale bar: 50 μm. Right: Quantification of TUNEL positivity using ImageJ (One way ANOVA). ***P<0.001; **P<0.01; *P<0.05.

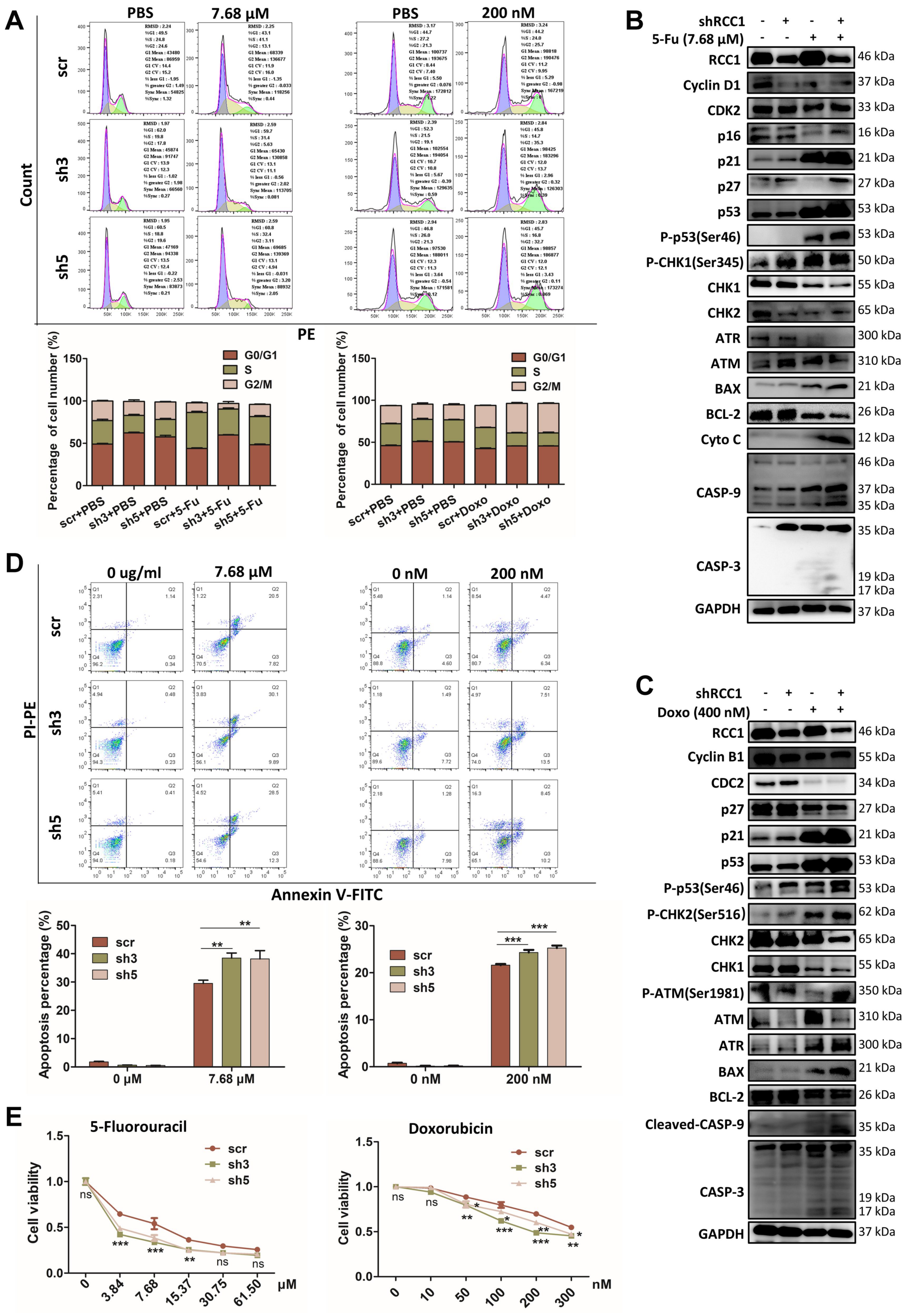

Supplementary Figure 4. RCC1 knockdown sensitizes CRC cell HCT116 to chemotherapy. (A) Cell cycle distribution in HCT116 cells was assessed by flow cytometry after 48-hour treatment with 7.68μM 5-FU or 200 nM Doxo for 48 h, with or without RCC1 knockdown. Statistical analysis of the proportion of cells in each

cell cycle phase was shown blow (Two-way ANOVA); (B and C) Western blot was performed to examine the expression of cell cycle-related and apoptosis-related proteins after treatment with 7.68 μM 5-FU or 400 nM Doxo. GAPDH was used as the loading controls; (D) Apoptosis in 5-FU/Doxo-resistant HCT116 cells after 48 h treatment with 7.68 μM 5-FU or 800 nM Doxo, with or without RCC1 knockdown. Statistical analysis of the proportion of cells in each apoptosis phase was shown blow (Two-way ANOVA); (E) Cell viability (CCK8) of HCT116 cells after 48 h treatment with various concentrations of 5-Fu or Doxo with or without RCC1 knockdown (Two-way ANOVA). ns, not significant; ****P*<0.001; ***P*<0.01; **P*<0.05.

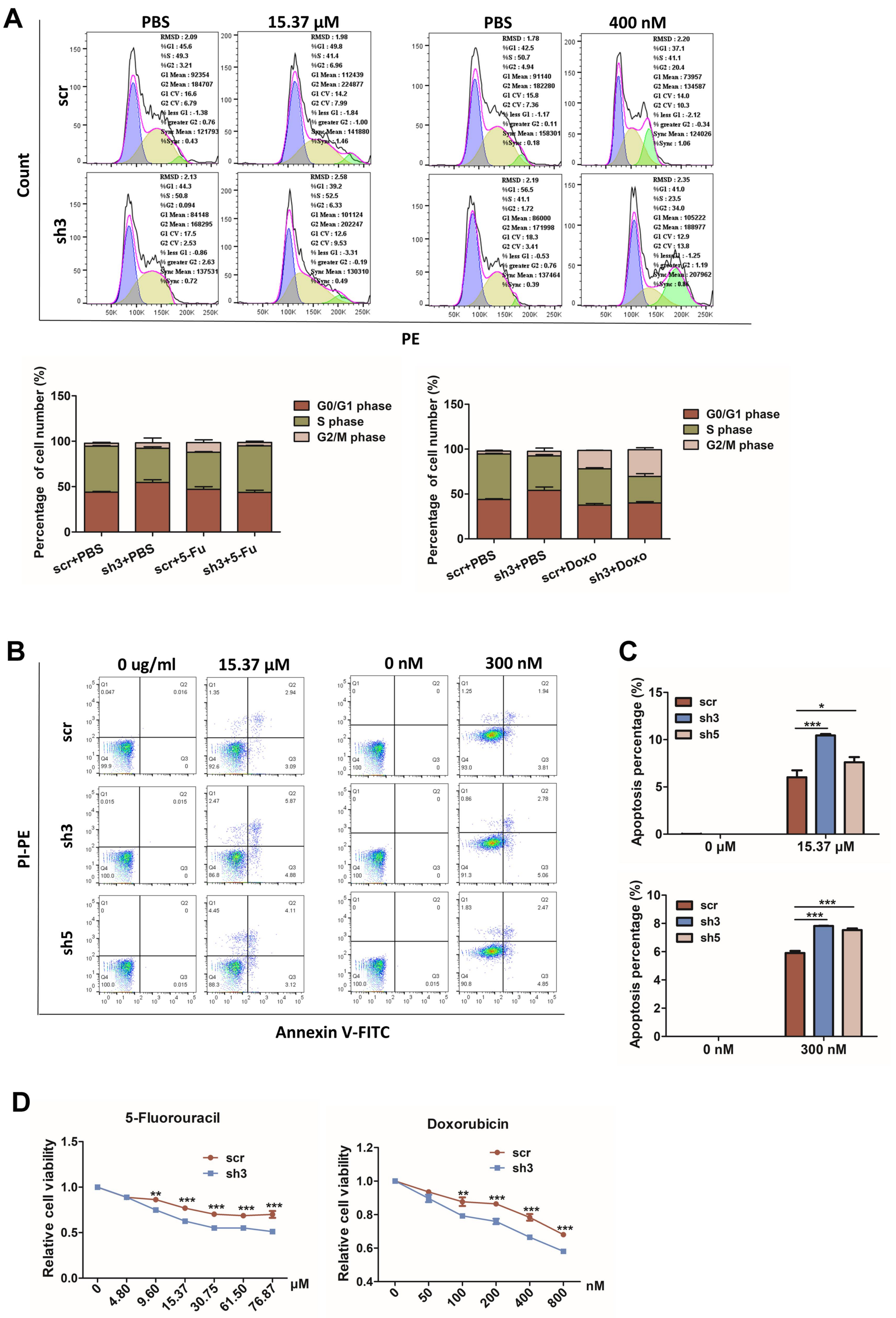

Supplementary Figure 5. RCC1 knockdown sensitizes CRC cell DLD-1 to chemotherapy. (A) Cell cycle distribution in DLD-1 cells was assessed by flow cytometry after 48-hour treatment with 15.37 μM 5-FU or 400 nM Doxo for 48 h, with or without RCC1 knockdown. Statistical analysis of the proportion of cells in

each cell cycle phase was shown blow (Two-way ANOVA); (B) Apoptosis in 5-FU/Doxo-resistant HCT116 cells after 48 h treatment with 15.37 μM 5-FU or 800 nM Doxo, with or without RCC1 knockdown; (C) Statistical analysis of apoptotic cell population (Two-way ANOVA); (D) Cell viability (CCK8) of HCT116 cells after 48 h treatment with various concentrations of 5-Fu or Doxo with or without RCC1 knockdown (Two-way ANOVA). GAPDH was used as the loading controls. ns, not significant; ***P<0.001; **P<0.01; *P<0.05.

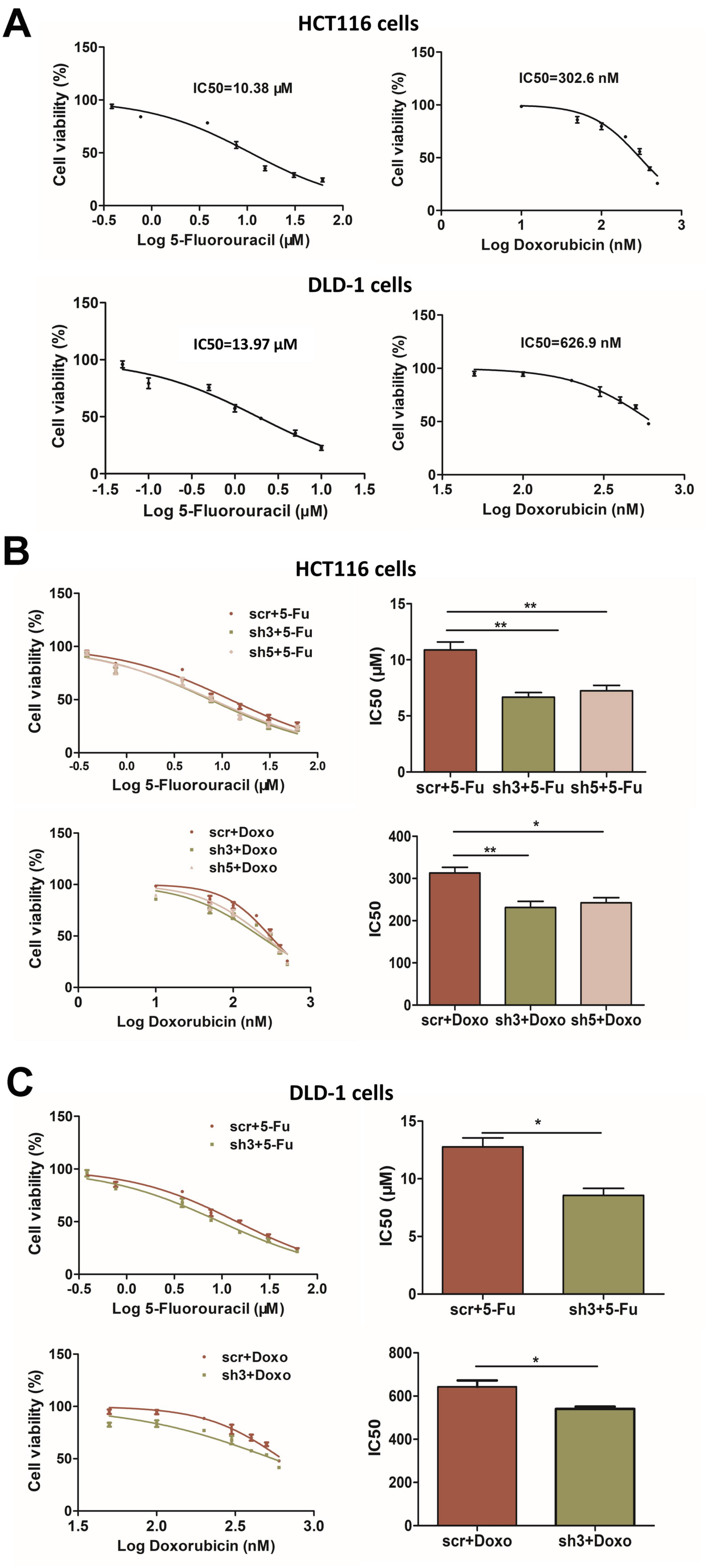
A
HCT116 cells
IC50=10.38 μM
Cell viability (%)
Log 5-Fluorouracil (μM)
IC50=302.6 nM
Log Doxorubicin (nM)
DLD-1 cells
IC50=13.97 μM
IC50=626.9 nM
B
HCT116 cells
scr+5-Fu
sh3+5-Fu
sh5+5-Fu
IC50 (μM)
scr+Doxo
sh3+Doxo
sh5+Doxo
IC50
C
DLD-1 cells
scr+5-Fu
sh3+5-Fu
scr+Doxo
sh3+Doxo

Supplementary Figure 6. (A) The IC_{50} of 5-FU and doxorubicin in HCT116 cells and DLD-1 cells; (B) the IC_{50} of 5-FU and doxorubicin in HCT116 cells following RCC1 knockdown (One way analysis of variance); (C) the IC_{50} of 5-FU and doxorubicin in DLD-1 cells following RCC1 knockdown (Two-tailed unpaired *t* test). ***P<0.001, **P<0.01, *P<0.05.

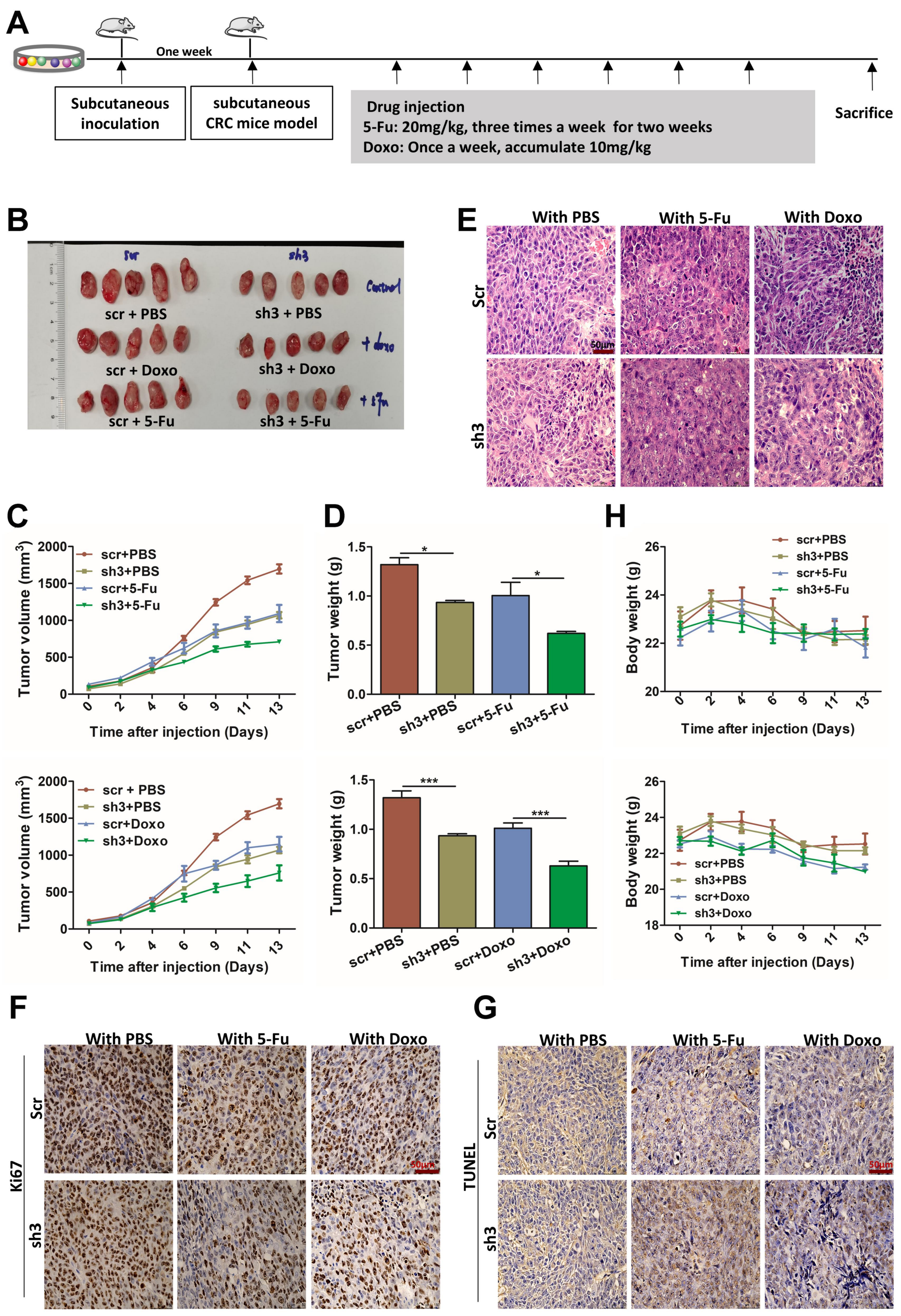

Supplementary Figure 7. RCC1 knockdown enhanced the inhibitory effect of 5-fluorouracil or doxorubicin on colorectal cancer *in vivo*. (A) Schematic overview of the experimental design: drug-resistant CRC cells were subcutaneously injected into nude mice, followed by systemic drug administration after tumor formation. Mice

were randomly divided into six groups, including scr + PBS (*n*=5), sh3+PBS (*n*=5), scr +5-Fu (*n*=5), sh3+5-Fu (*n*=5) (20mg/kg, 3×/weeks,2 weeks), scr +doxo (*n*=5), scr +doxo (*n*=5) (10 mg/kg cumulative, 1×/week); (B) Representative images of excised subcutaneous tumors in the CRC model after treatment; (C) Tumor volume measurements during treatment; (D) Final tumor weights across all groups (One-way ANOVA); (E) Representative H&E staining of tumor sections from each group. Scale bar: 50 μm; (F) Representative images of Ki-67 immunohistochemistry. Scale bar: 50 μm; (G) Representative images of TUNEL staining across groups. Scale bar: 50 μm; (H) Body weight monitoring of mice during treatment to assess tolerability (Two-way ANOVA). ***$P<0.001$; **$P<0.01$; *$P<0.05$.

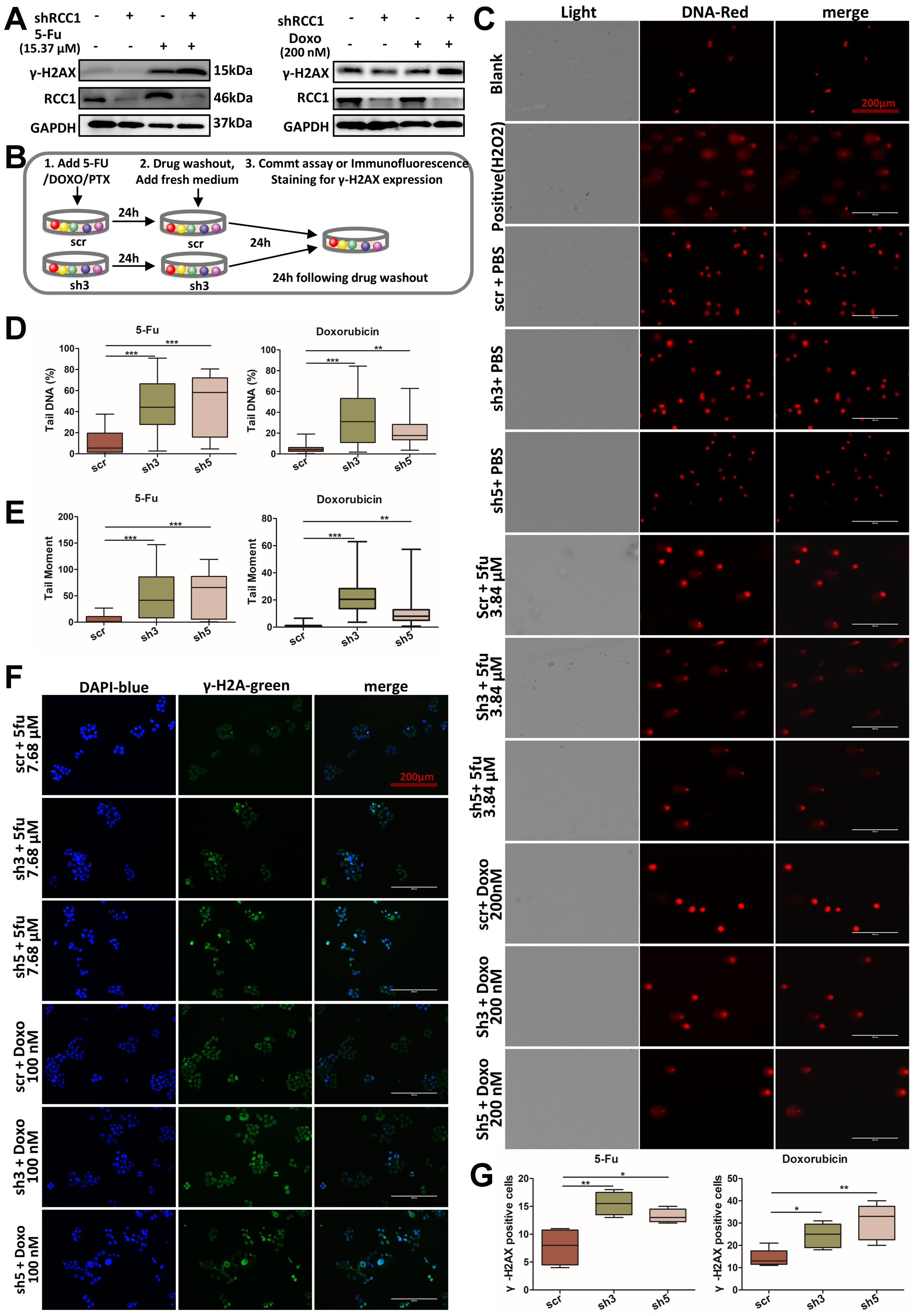

Supplementary Figure 8. RCC1 knockdown enhances DNA damage and impairs DDR in CRC cells. (A) Western blot analysis of γ-H2AX expression in drug-resistant CRC cells treated with 15.37 μM 5-FU or 200 nM Doxo for 48 h, with or without RCC1 knockdown for 48 h; (B) Schematic diagram illustrating the experimental

design for monitoring DNA damage repair dynamics *via* γ-H2AX immunofluorescence at 24 h post drug withdrawal; (C) Representative comet assay images of drug-resistant CRC cells treated with the indicated drugs for 24 h. Scale bar: 100 μm; (D and E) Quantification of DNA damage based on comet assay, including percentage of DNA in tail (D) and tail moment (E) across different treatment groups (One-way ANOVA). Scale bar: 200 μm; (F) Representative immunofluorescence images showing time-dependent changes in γ-H2AX-positive cells after treatment with 7.68 μM 5-FU or 100 nM Doxo in CRC cells with or without RCC1 knockdown. Scale bar: 50 μm; (G) Quantification of γ H2AX-positive cells at 24 h after drug removal in the indicated groups, highlighting persistent DNA damage in the RCC1 knockdown condition (Two-way ANOVA). ns, not significant; ***P<0.001; **P<0.01; *P<0.05.
